# Immunomodulatory Peptides as Vaccine Adjuvants and Antimicrobial Agents

**DOI:** 10.3390/ph17020201

**Published:** 2024-02-02

**Authors:** Shiva Hemmati, Zahra Saeidikia, Hassan Seradj, Abdolali Mohagheghzadeh

**Affiliations:** 1 Department of Pharmaceutical Biotechnology, School of Pharmacy, Shiraz University of Medical Sciences, Shiraz 71345-1583, Iran; 2 Biotechnology Research Center, Shiraz University of Medical Sciences, Shiraz 71345-1583, Iran; 3 Department of Pharmaceutical Biology, Faculty of Pharmaceutical Sciences, UCSI University, Cheras, Kuala Lumpur 56000, Malaysia; 4 Student Research Committee, Shiraz University of Medical Sciences, Shiraz 71345-1583, Iran; z.saeidikia@gmail.com; 5 Department of Medicinal Chemistry, School of Pharmacy, Shiraz University of Medical Sciences, Shiraz 71345-1583, Iran; serajh@sums.ac.ir; 6 Department of Phytopharmaceuticals, School of Pharmacy, Shiraz University of Medical Sciences, Shiraz 71345-1583, Iran; mohaghegh@sums.ac.ir

**Keywords:** adjuvant, antibiotics, antigen-presenting cell, antimicrobial peptide, anticancer peptide, artificial intelligence, cell-penetrating peptide, cytokine, immunotherapy

## Abstract

The underdevelopment of adjuvant discovery and diversity, compared to core vaccine technology, is evident. On the other hand, antibiotic resistance is on the list of the top ten threats to global health. Immunomodulatory peptides that target a pathogen and modulate the immune system simultaneously are promising for the development of preventive and therapeutic molecules. Since investigating innate immunity in insects has led to prominent achievements in human immunology, such as toll-like receptor (TLR) discovery, we used the capacity of the immunomodulatory peptides of arthropods with concomitant antimicrobial or antitumor activity. An SVM-based machine learning classifier identified short immunomodulatory sequences encrypted in 643 antimicrobial peptides from 55 foe-to-friend arthropods. The critical features involved in efficacy and safety were calculated. Finally, 76 safe immunomodulators were identified. Then, molecular docking and simulation studies defined the target of the most optimal peptide ligands among all human cell-surface TLRs. SPalf2-453 from a crab is a cell-penetrating immunoadjuvant with antiviral properties. The peptide interacts with the TLR1/2 heterodimer. SBsib-711 from a blackfly is a TLR4/MD2 ligand used as a cancer vaccine immunoadjuvant. In addition, SBsib-711 binds CD47 and PD-L1 on tumor cells, which is applicable in cancer immunotherapy as a checkpoint inhibitor. MRh4-679 from a shrimp is a broad-spectrum or universal immunoadjuvant with a putative Th1/Th2-balanced response. We also implemented a pathway enrichment analysis to define fingerprints or immunological signatures for further in vitro and in vivo immunogenicity and reactogenicity measurements. Conclusively, combinatorial machine learning, molecular docking, and simulation studies, as well as systems biology, open a new opportunity for the discovery and development of multifunctional prophylactic and therapeutic lead peptides.

## 1. Introduction

Irrational prescriptions, incorrect diagnoses, easy access, overuse, prophylaxis, and insufficient doses for antibiotic consumption have led to the development of drug-resistant microbial species [1,2]. Antibiotic resistance can be classified into intrinsic, acquired, and adaptive forms [3]. For example, the impermeability of the outer membrane of Gram-negative bacteria to large polar antibiotics or the lack of a target are intrinsic resistance mechanisms. Antibiotic inactivation, alteration in the target, activation of efflux pumps, and reduction of uptake are the main mechanisms of acquired antibiotic resistance. Microorganisms’ main adaptive antibiotic resistance is via the development of biofilm polymeric matrices and conversion to persister cells with slow growth [4,5]. Notably, the borders between these mechanisms are sometimes blurred, and a mechanism such as efflux pump activation can be intrinsic or acquired. Without adequate action, the world will face 10 million deaths annually due to antimicrobial resistance by 2050 [6].

On the other hand, current vaccination platforms have limitations in the provision of long-term protection, inadequate immunity in aged populations, and an inability to induce efficient cellular immunity [7]. Adjuvants are incorporated into the formulation of vaccines to boost the potency, spectrum, and durability of immune responses [8]. The underdevelopment of adjuvant discovery and diversity, compared to core vaccine technology, is evident [9]. To obtain approval for clinical use, adjuvants must meet four fundamental conditions. They should induce a potent cellular and humoral response and lead to long-term immunity. Regarding safety conditions, adjuvants should be nontoxic without causing autoimmune or allergic reactions [10]. The low diversity of vaccine adjuvants may originate from the fact that adjuvants are not developed for specific illness conditions, and a general adjuvant is incorporated in all formulations. From the viewpoint of pharmaceutical companies, the selection of an adjuvant for a new vaccine is a business decision, as the risk of using a new adjuvant may appear heavier than the benefit of a conventional adjuvant with a firm clinical track record [11]. So far, the FDA has approved fewer than ten adjuvants [12]. A timeline analysis shows that improved immunization by adding adjuvants to vaccine formulations has a history going back approximately one century, when Glenny et al., in 1926, precipitated antigens on alum particles [13,14]. Despite the strong induction of a humoral response, weak cellular immunity is provoked by aluminum salts. After, in 1997, squalene emulsion-based MF59 (oil-in-water) and adjuvant systems (AS) received approval for human vaccines. For example, monophosphoryl lipid A (MPL), a safe derivative of LPS, acts as a toll-like receptor 4 (TLR4) agonist in AS01, AS02, and AS04 adjuvants [9]. The COVID-19 pandemic has reignited the need to invest in strategic vaccine design and delivery approaches, leading to the licensing of lipid nanoparticles (LNPs). LNPs are delivery systems that can act as adjuvants [15]. However, delivery systems only promote antigen presentation by MHCs and do not affect the cytokine or other costimulatory signaling pathways.

**Figure 1:**
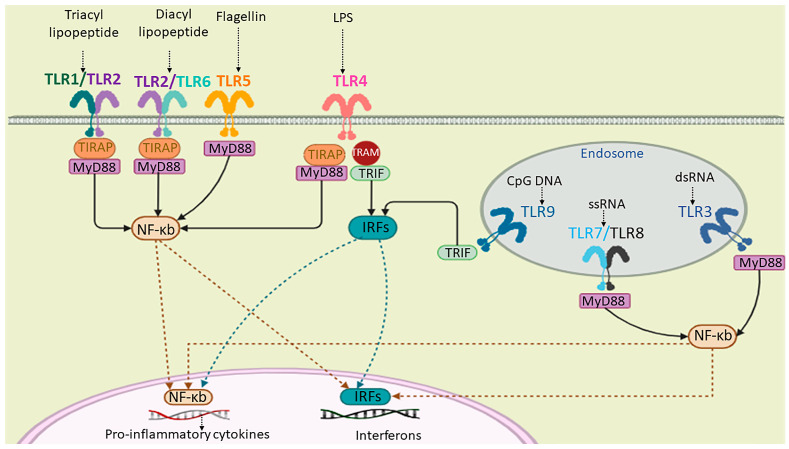
A schematic representation of human TLRs’ (a group of PRRs) localization, their natural ligands, and signaling. TLR1, TLR2, TLR4, TLR5, and TLR6 are cell-surface receptors and TLR3, TLR7, TLR8, and TLR9 are localized on the endosomal membrane. The extracellular domain of TLRs binds to the corresponding PAMP or DAMP ligands. TLRs can dimerize through their cytoplasmic toll/interleukin-1 receptor/resistance protein (TIR) domain. The activation of TLR signaling is dependent on the myeloid differentiation primary response protein 88 (myD88) or TIR-domain-containing adapter-inducing interferon-β (TRIF) signaling cascades, which, in turn, results in the activation of interferon regulatory factors (IRFs) and NF-kb for the production of cytokines.

With more than 80 FDA-approved peptide therapeutics, peptides are known as highly selective, specific, and biocompatible medications [16]. The capacity of immunomodulatory peptides with adjuvanticity properties (i.e., immunoadjuvants) has been extended recently in such a way that they not only boost the immune system but also display antimicrobial and antitumor activities themselves [17]. In the presence of immunomodulatory peptides that act as adjuvants, the maturation of a higher number of antigen-presenting cells (APCs), which elevates APC crosstalk with T-cells, is observed. This communication generates multifunctional T-cells and various classes of cytokines and antibodies [18]. In other words, adjuvants train adaptive immunity by stimulating innate immune cells and triggering pattern recognition receptor (PRR) signaling. These immunostimulatory adjuvants, such as pathogen-associated molecular patterns (PAMPs), damage-associated molecular patterns (DAMPs), and TLR agonists, elevate antigen presentation on MHCs, induce costimulatory molecules, such as clusters of differentiation (CD) on APCs, and express secretion of cytokines [19] (Figure 1). The production of neutralizing antibodies and cytotoxic T-cells (CTLs) against defined antigens induces immune system memory cells for durable protection against infection during the vaccination process [9].

The development of broad-spectrum peptides as pan-antimicrobials and vaccine adjuvants has been reported previously. For example, the scope of granulocyte-colony stimulating factor (GCSF) activity ranges from vaccine adjuvant to antiviral immunotherapy [20]. Human defensins are host defense peptides (HDPs) or antimicrobial peptides (AMPs) that target pathogens and modulate the immune system concurrently, proposed as vaccine immunoadjuvants [21]. In parallel to modulating the immune system, AMPs disrupt membrane permeation and might interfere intracellularly with microbial transcription and translation processes [22]. AMPs, such as LL-37 derived from human cathelicidin, are HDPs with antimicrobial and immunomodulating properties linking innate and adaptive immunities [23]. A similar concept exists for developing cancer vaccine adjuvants with the extra ability to target tumor cells and which is applicable for cancer immunotherapy [24]. Safe and immunogenic MPL-adjuvanted vaccines are TLR4 agonists and have also been applied in cancer vaccines [25].

Although AMPs can induce the immune system, short immunomodulatory peptides are considered effective, safe, and economically feasible adjuvants for next-generation vaccine design [26]. Improvements in high throughput technologies, such as omics studies in parallel with machine learning and deep learning approaches, provide a rich pool of natural leads for drug discovery [27]. Invertebrates such as arthropods lack adaptive immunity and depend exclusively on innate immunity to defend themselves [28]. The slow rate of adjuvant discovery in humans originates from a strategy mainly based on adaptive immunity induction to trigger immunologic memory, ignoring other critical immunological elements required to boost vaccine efficacy. Favorably, considering innate immunity induction, which forms the adaptive immune reaction, has renovated the adjuvant’s mechanism of action [29].

Within this study, we investigated the most potent immunomodulating cryptic peptide fragments inside AMPs derived from arthropods using combinatorial machine learning, docking, simulation, and systems biology studies. We introduce immunoadjuvant peptides that target the pathogens or tumor cells as antimicrobial and anticancer agents in addition to their potency for inducing APCs and immune system stimulation. Because in vitro and in vivo adjuvanticity results are usually uncorrelated [11], we propose a so-called “systems adjuvantology” approach by identifying pathways and biological processes targeted by the identified adjuvants. These multifunctional peptides are a novel paradigm in peptide discovery and open a new trend in developing adjuvants and antibiotics.

## 2. Results

### 2.1. Retrieval of Cryptic Immunomodulatory Peptides from AMPs in Arthropods

A total of 643 AMPs of diverse arthropod species were retrieved from the InverPep database (Table 1). Employing the VaxinPAD program, encrypted 10-mer and 15-mer peptides with immunomodulatory properties were extracted. Subsequently, immunomodulatory peptides with a score higher than 0.7 that were predicted to be nontoxic, nonallergenic, and nonhemolytic with no propensity to aggregation, known as “safe peptides”, were included for further analyses. The data reduction process identified 76 immunomodulatory peptides that met the efficacy and safety criteria (Supplementary Materials S1 and S2).

### 2.2. Identification of Immunomodulatory Peptides with Antimicrobial and Anticancer Properties

To identify bifunctional peptides with concurrent properties in pathogen targeting and immune system modulation, MetaiAVP, AntiFP, and antiTBpred programs were used to define putative antiviral, antifungal, and antitubercular peptides with immunomodulatory properties out of the pool of 76 final candidates. Immunomodulatory peptides with anticancer characteristics were also determined (Supplementary Materials S2). Finally, immunomodulators with the highest SVM score and the most potent antimicrobial or anticancer properties, possessing acceptable physiochemical characteristics, such as an appropriate isoelectric point (pI) and stability in an aqueous environment, were selected as the most optimal candidates to find the target TLR (Table 2). The most potent immunomodulator with concomitant strong antitubercular, antibacterial, and antiangiogenic properties was named the universal adjuvant.

### 2.3. Docking Bifunctional Immunomodulatory Peptides with TLRs

TLRs can be named the “Swiss Army” knife of immunity, prepared to react to numerous disease states. The most optimal immunomodulatory peptides that are reported in Table 2 were subjected to molecular docking analysis with all hTLRs, including TLR1/TLR2, TLR2, TLR2/TLR6, TLR4/MD2, TLR5, and TLR6, using the ClusPro 2.0 program. This aimed to identify the target with the most stable peptide–receptor complexes. The SPalf2-453 (HIRRRPKFRK) ligand, a decameric cationic antiviral peptide with strong immunomodulatory potency, was sourced from the antilipopolysaccharide factor (ALF-2), an AMP found in a mud crab *Scylla paramamosain*. Compared to a 35-mer antiviral peptide as the positive control, namely, An1a from spider (GFGCPLDQMQCHNHCQSVRYRGGYCTNFLKMTCKCY), SPalf2-453 displayed stronger immunomodulatory and comparable antiviral potency. While SPalf2-453 showed immunomodulatory and antiviral scores of about 1.04 and 0.998, respectively, An1a had immunomodulatory and antiviral scores of about 0.55 and 0.92. With a score of −1171.5, the TLR1/TLR2 heterodimer was the most optimal target for SPalf2-453 according to the ClusPro 2.0 program.

The LSsty1-174 ligand, a 10-mer peptide sequence “PCVQQPCPKC” derived from *Litopenaeus stylirostris*, a shrimp of the Penaeidae family, exhibits immunomodulatory and antifungal properties. With a score of −1408.8, TLR2 was the best target for LSsty1-174 using the ClusPro2.0 tool. The PPpp113-266 peptide (RVQERRFKRI), derived from PP113 AMP of an endoparasitic wasp (*Pteromalus puparum*), displays immunomodulatory and antitubercular properties. With a score of −1220, TLR2 was the most optimal target receptor for LSsty1-174. The SBsib-711 ligand (KLKRGAKKAL) derived from SibaCec, a cecropin-like antimicrobial peptide in the salivary gland of the black fly *Simulium bannaense* has both immunomodulatory and anticancer properties. With a ClusPro score of −1043, TLR4/MD2-SBsib-711 was the most optimal complex compared to other ligands with anticancer characteristics. Finally, the most optimal universal immunoadjuvant was MRh4-679 (KPAIRRLARR) from a core histone 4 protein in *Macrobrachium rosenbergii* shrimp. TLR4/MD2 was the best target for MRh4-679, with a ClusPro score of −1642.6. The physiochemical characteristics, cytokine induction ability, and cell-penetration potency of these five optimal candidates are collected in Table 2, and detailed docking analyses are reported in the following sections.

### 2.4. TLR4/MD2-WALK244.04 Complex as the Positive Control

**Figure 2:**
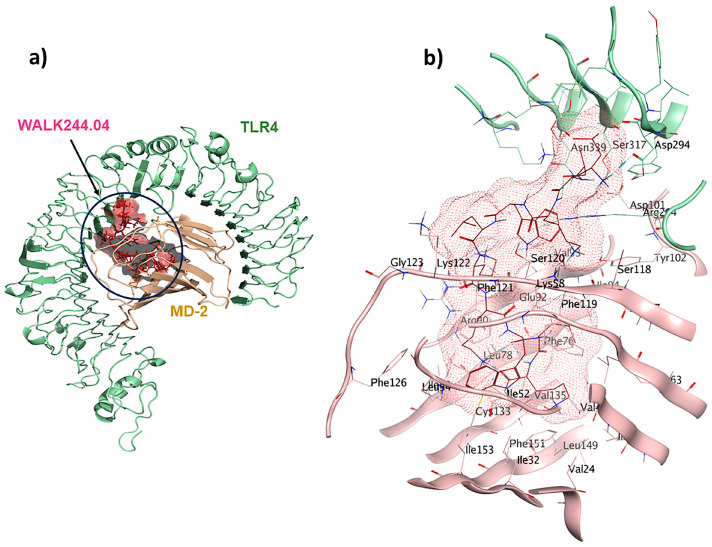
A comprehensive overview of the positive control (WALK244.04) interactions with its receptor: (**a**) TLR4/MD2-WALK244.04 docked complex; (**b**) 3D binding pocket interactions.

WALK244.04 is a synthetic 10-mer peptide (LLKWLKKWLK) with dual antimicrobial and anti-inflammatory properties. This peptide was strategically designed with a focus on WALK, an acronym signifying tryptophan-containing amphipathic-helical leucine/lysine peptides. These peptides emulate the cationic, amphipathic α-helical structures found in antimicrobial peptides, utilizing only three types of amino acids [31]. In our study, WALK244.04 was used as the positive control and underwent molecular docking simulations as the ligand for the TLR4/MD2 receptor. In this context, we designated Chain B (comprising R264, V316, S317, N339, and S360) and Chain D (comprising K91, E92, I94, C95, R96, S98, D99, D100, D101, Y102, and C105) as the binding site regions via MOE SiteFinder and employed rigid docking methodologies. Chain B corresponds to TLR4, whereas Chain D represents MD2. The MOE docking evaluated the ligand–receptor binding energy of approximately −25.2 kcal/mol. The positive control ligand formed ten interactions within the TLR4/MD2-WALK244.04 complex (Figure 2). Of these, five interactions were observed within the hydrophobic pocket of the coreceptor (MD2), and the remaining five interactions with TLR4 as the main receptor. Four of these interactions are characterized by hydrogen bonds, each with distances falling within the 2.7 to 3.3 Å range. Glu92 contributes to forming one hydrogen bond as a side-chain H donor and participates in two ionic interactions due to its acidic and polar nature. Additionally, Phe119 forms a hydrogen bond as a backbone donor, while Arg264 participates in two π-cation interactions. Asp294, with its acidic nature, contributes to forming a hydrogen bond as a side-chain donor, and Cys133 is involved in creating a hydrogen bond as a side-chain acceptor (Figure 2).

**Figure 3:**
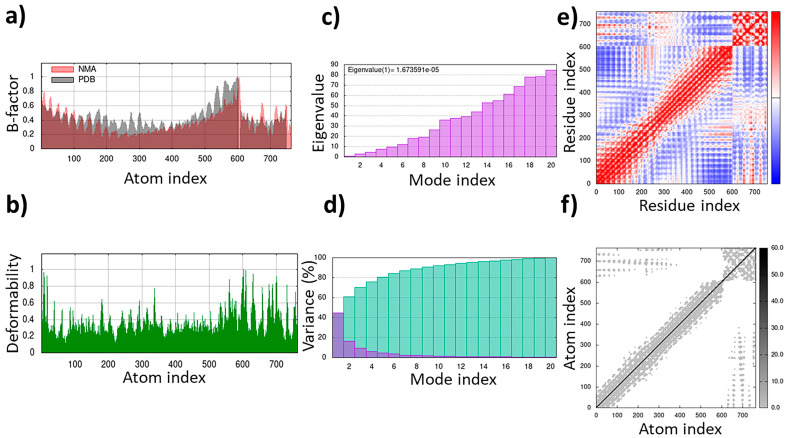
The results of the molecular dynamics simulation of the TLR4/MD2-WALK244.04 complex as the positive control: (**a**) B-factor; (**b**) deformability; (**c**) eigenvalues; (**d**) variance; (**e**) covariance matrix; (**f**) elastic network map.

The TLR4/MD2-WALK244.04 complex underwent MD simulations for stability evaluation using the iMDOS webserver. The analysis employed normal mode analysis (NMA) through the iMODS approach, which characterizes macromolecular functional motions based on relative motion extent frequencies and deformation vectors. This method provides insight into molecular flexibility in a cellular context. The MD simulation result for the TLR4/MD2-WALK244.04 complex is depicted in Figure 3. The B-factor graph links the mobility of the NMA-docked complex to PDB scores, reflecting the average root mean square deviation (RMSD) (Figure 3). The deformability graph identifies high deformability peaks within the protein, typically representing flexible regions with elevated values and rigid segments with lower values in the primary chain residues [32,33]. Each normal mode is associated with a unique eigenvalue characterizing the molecular rigidity. A lower eigenvalue implies less energy required for structural deformation, demonstrating stability. For the TLR4/MD2 and WALK244.04 complex, the eigenvalue was approximately 1.673591e-05. The variance graph, inversely related to eigenvalue, displays individual and cumulative variance. The covariance matrix indicates residue correlations, classified as correlated (red), uncorrelated (white), or anticorrelated (blue), with stronger correlations indicating a well-structured complex. The elastic network model outlines atomic connections, with darker points denoting stiffness and lighter points representing flexibility (Figure 3).

### 2.5. TLR1/TLR2-SPalf2-453 as an Antiviral Immunomodulator

**Figure 4:**
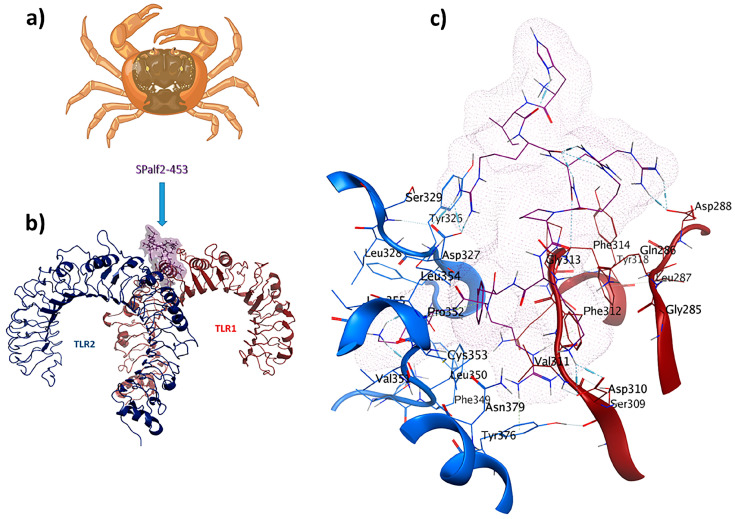
SPalf2-453 (HIRRRPKFRK) is a crab-derived immunomodulator with antiviral properties: (**a**) *Scylla paramamosain*; (**b**) comprehensive overview of TLR1/TLR2-SPalf2-453 docked complex; (**c**) ligand–receptor 3D interactions in the binding pocket.

SPalf2-453 (HIRRRPKFRK) was identified as a TLR1/TLR2 receptor ligand. An MOE dock was employed to analyze the binding interactions for the most optimal peptides. Chain A (comprising F322, Y323, L324, F325, Y326, D327, F349, L350, and P352) and Chain B (comprising F312, G313, F314, P315, and Q316) were selected as the binding site regions. Chain A corresponds to TLR2, whereas Chain B represents TLR1. This re-docking effort yielded a −72.1 kcal/mol binding energy. Interaction details in the binding pocket are shown in Figure 4. The antiviral ligand SPalf2-453 establishes 20 interactions within the TLR1/TLR2-SPalf2-453 complex. These interactions are evenly distributed, wherein ten interactions are observed with TLR1, and the remaining ten linkages are observed with TLR2. Half of these interactions consist of hydrogen bonds, signifying their structural stability, with distances falling within the 2.7 to 3.3 Å range. Asp327 plays an important role, forming three hydrogen bonds as a side-chain donor and engaging in four ionic interactions as a backbone acceptor, owing to its acidic and polar characteristics. Furthermore, Phe314 is a backbone acceptor in hydrogen bonding, while Leu350 acts as a backbone donor in a hydrogen bond interaction with TLR2. The TLR1/TLR2-SPalf2-453 showed a well-structured complex in MD simulation results with an eigenvalue of about 1.061859e-05, aligned with the positive control (Supplementary Materials S3).

### 2.6. Antitubercular and Antifungal Immunomodulators

**Figure 5:**
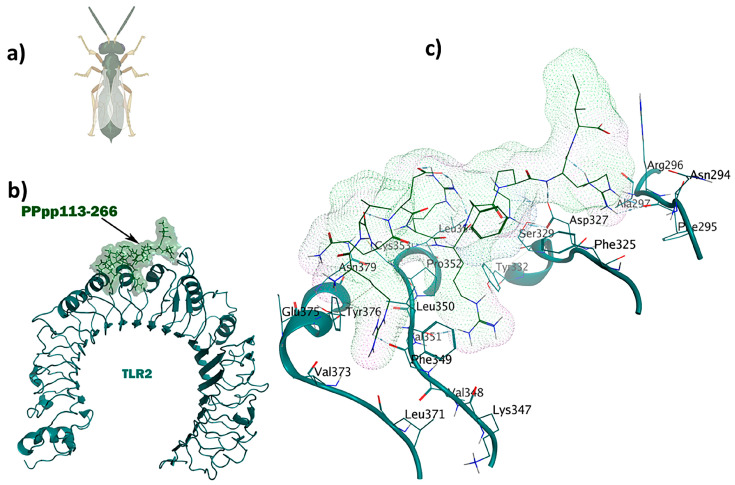
The PPpp113-266 ligand (RVQERRFKRI) derived from the wasp shows simultaneous immunomodulatory and antitubercular properties: (**a**) *Pteromalus puparum*; (**b**) visualization of the TLR2-PPpp113-266 docked complex; (**c**) 3D interactions of the ligand–receptor in the binding pocket.

The wasp-derived peptide, PPpp113-266 (RVQERRFKRI), showed antitubercular properties. Compared to the brevinin-derived antitubercular peptide used as the positive control [34] with a score of 0.26, PPpp113-266 displayed much stronger antitubercular activity. The TLR2-PPpp113-266 complex exhibited significant stability with a binding energy score of −49.6 kcal/mol using MOE (Figure 5). The binding site was selected within Chain A, representing TLR2 (L266, L289, L312, I314, L317, I319, Y323, F325, Y326, D327, L328, S329, L331, Y332, T335, I341, V343, S346, K347, V348, F349, L350, V351, P352, L355, L359, L365, and L367). Out of a total of 16 interactions, seven H bonds were observed. Interestingly, because of its polar and acidic characteristics, Asp327 was involved in the formation of three hydrogen and three ionic bonds. Arg296 contributed to the formation of five ionic interactions. Both Phe349 and Leu350 were involved in the formation of a side-chain H bond. Tyr376 participated through an H-π interaction in the complex.

**Figure 6:**
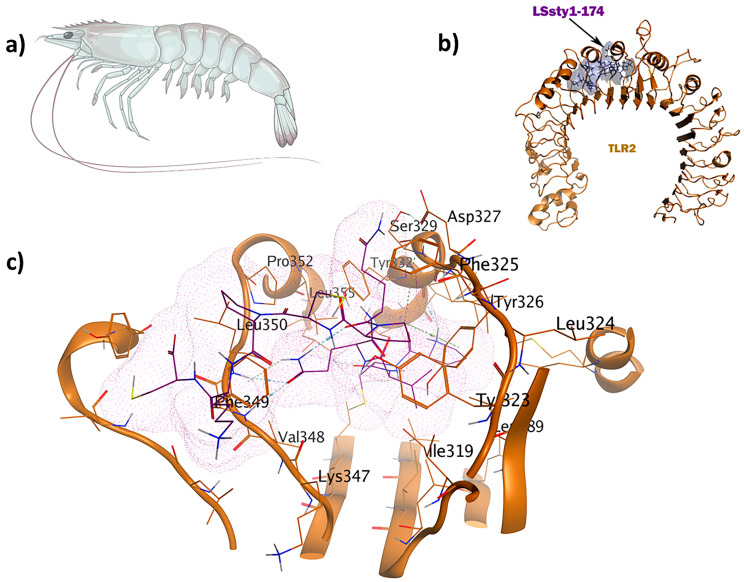
The LSsty1-174 ligand (PCVQQPCPKC) derived from shrimp has immunomodulatory and antifungal properties: (**a**) *Litopenaeus stylirostris*; (**b**) visualization of TLR2-LSsty1-174 docked complex; (**c**) 3D interactions of the ligand–receptor in the binding pocket.

The shrimp-derived peptide LSsty1-174 (PCVQQPCPKC) acts as an immunomodulator with antifungal properties. The antifungal peptide known as Ar-AMP was used as the positive control, which showed mild antifungal (0.32) and immunomodulatory (0.30) properties compared to LSsty1-174 [35]. Re-docking was carried out, employing the same binding site used for the antitubercular candidate, as their receptors were identical. The TLR2-LSsty1-174 complex shows seven interactions in the binding site (Figure 6). Phe349 is involved in a hydrogen bond as a backbone H acceptor and an arene-H interaction. Leu350 and Phe325 contribute to H bonds and arene-H interactions, respectively. Tyr326 displays two π-H interactions. MD simulation studies show that both TLR2-PPpp113-266 and TLR2-LSsty1-174 are well-structured and stable complexes with eigenvalues of approximately 1.518058e-05 and 1.819793e-05, respectively, aligning with the positive control (Supplementary Materials S3).

### 2.7. TLR4/MD2-SBsib-711 as an Anticancer Immunomodulator

**Figure 7:**
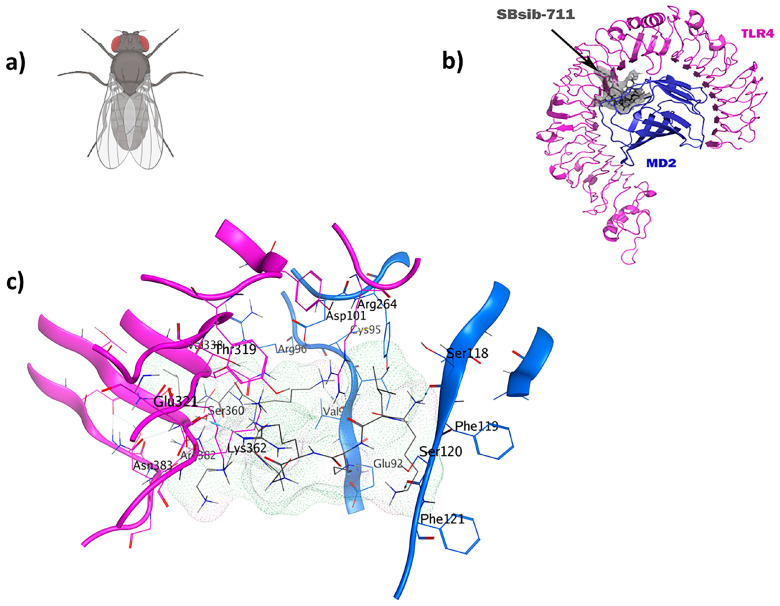
The SBsib-711 ligand (KLKRGAKKAL) from a black fly has immunomodulatory and anticancer characteristics: (**a**) *Simulium bannaense*; (**b**) visualization of the TLR4/MD2-SBsib-711 docked complexes; (**c**) ligand–receptor 3D interactions in the binding pocket.

**Figure 8:**
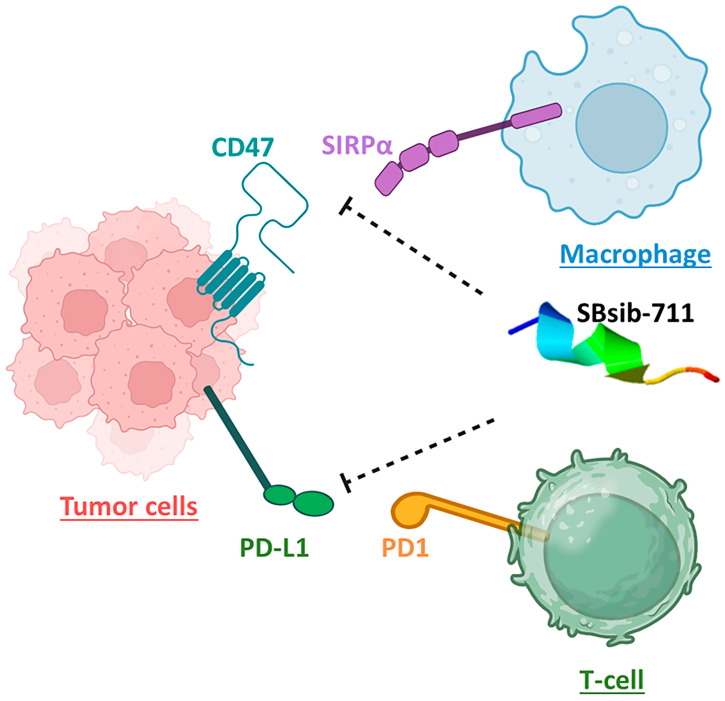
The anticancer peptide SBsib-711 is putatively an immune checkpoint inhibitor. It binds CD47 and PD-L1 on tumor cells and prevents CD47/SIRPα and PD1/PD-L1 interactions allowing the macrophage and activated T-cells to attack tumor cells, respectively.

The blackfly-derived peptide SBsib-711 (KLKRGAKKAL) has immunomodulatory and anticancer properties. In the TLR4/MD2-SBsib-711 complex, we designated Chain B (comprising R264, V316, S317, N339, and S360) and Chain D (comprising K91, E92, I94, C95, R96, S98, D99, D100, D101, Y102, and C105) as the binding site regions via MOE SiteFinder. Chain B corresponds to TLR4, whereas Chain D represents MD2 (Figure 7). At least six hydrogen bonds were observed, all within a 2.7–3.3 Å distance. Similar to the positive control and in line with previous studies, Ser118 and Ser120 formed H bonds. Glu92 contributes to the formation of one hydrogen bond as a side-chain H donor and participates in one ionic interaction due to its acidic and polar nature. Val93 is involved in forming a hydrogen bond as a backbone H donor. Arg264 contributes to forming a hydrogen bond as a side-chain H acceptor. MD simulation studies show that TLR4/MD2-SBsib-711 is a stable complex with an eigenvalue of approximately 1.974798e-05 (Supplementary Materials S3). The PDL1Binder program [36] predicted SBsib-711 as a putative ligand for programmed death ligand-1 (PDL-1); hence, it can block PD-1/PD-L1 interactions. According to the prediction of the CD47Binder program [37], SBsib-711 is a strong binder to CD47 (score 0.86) and potentially prevents CD47 interaction with SIRPα (Figure 8).

### 2.8. TLR4/MD2-MRh4-679 Complex as the Universal Immunoadjuvant

**Figure 9:**
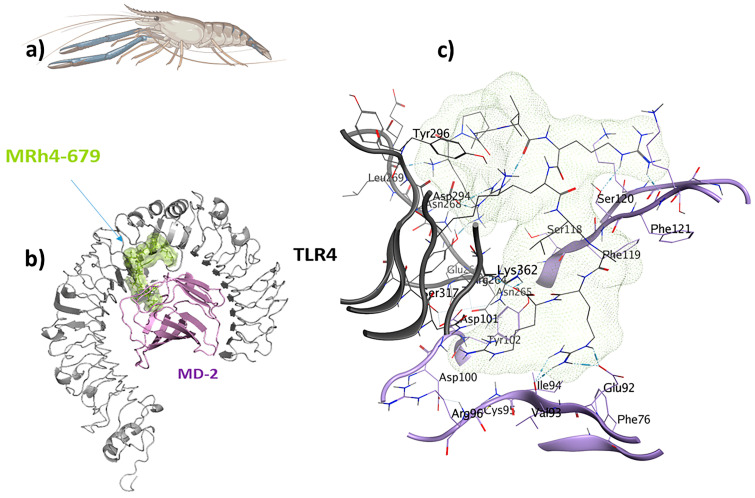
The MRh4-679 ligand (KPAIRRLARR) originated from a shrimp with immunomodulatory, antiviral, antifungal, antitubercular, and antiangiogenic properties and is suitable as a universal immunoadjuvant: (**a**) *Macrobrachium rosenbergii*; (**b**) visualization of the TLR4/MD2-MRh4-679 docked complex; (**c**) ligand–receptor 3D interactions in the binding pocket.

The shrimp-derived peptide MRh4-679 (KPAIRRLARR) displayed immunomodulatory, antiviral, antifungal, antitubercular, and anticancer properties. A docking analysis was performed for TLR4/MD2-MRh4-679 by designating Chain B (comprising R264, V316, S317, N339, and S360) and Chain D (comprising K91, E92, I94, C95, R96, S98, D99, D100, D101, Y102, and C105) as the binding site regions. Chain B corresponds to TLR4, whereas Chain D represents MD2 (Figure 9). This re-docking effort yielded a binding energy score of −70.3 kcal/mol.

**Figure 10:**
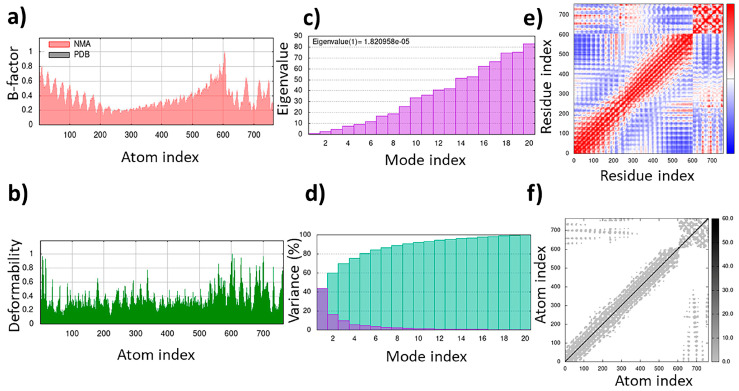
Molecular dynamics simulation of the TLR4/MD2-MRh4-679 complex as the universal immunoadjuvant: (**a**) B-factor; (**b**) deformability; (**c**) eigenvalues; (**d**) variance; (**e**) covariance matrix; (**f**) elastic network map.

A total of 24 interactions were observed within the TLR4/MD2-MRh4-679 complex (Table 3). Of these, 13 interactions were located within the hydrophobic pocket of the coreceptor (MD2), while the remaining 11 resided in TLR4. Twelve interactions were in the form of hydrogen bonds, showing their stability with distances ranging from 2.7 to 3.3 Å. An analysis of the TLR4/MD2-MRh4-679 complex shows that Glu92 is involved in the formation of two hydrogen bonds as a backbone H donor and two ionic interactions, characterized by its acidic and polar properties. Additionally, Ser120 serves as a side-chain donor in one hydrogen bond, and Phe121 contributes as a backbone donor in a hydrogen bond. Lys362 and Arg264, both as cationic residues, are involved in forming hydrogen bonds as side-chain acceptors. Arg264 also participates in two ionic interactions. Asp294, marked by its acidic nature, forms two hydrogen bonds as a side-chain H donor, along with five ionic bonds. Val93 contributes by forming two hydrogen bonds as a backbone donor, while Asp101 contributes to the formation of three ionic interactions. Detailed information on interactions is provided in Table 3. The TLR4/MD2-MRh4-679 complex showed a high residue correlation, atomic connection, and a well-structured complex in the MD simulation, with an eigenvalue of approximately 1.820958e-05, aligning with the positive control (Figure 10).

### 2.9. Evaluating the In Vivo Targets of the Identified Immunoadjuvants by Systems Biology

**Figure 11:**
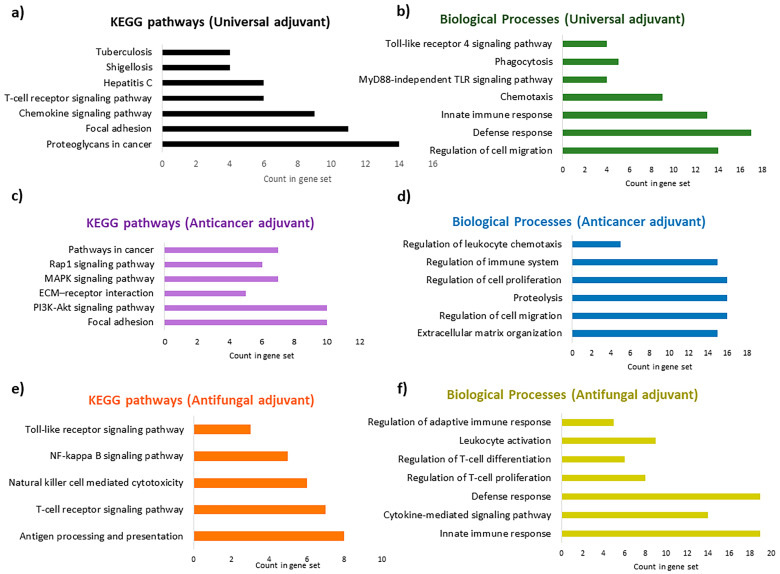
Enriched Gene Ontology (GO) terms for biological processes (BPs) and KEGG pathways for the most optimal adjuvants: (**a**) enriched KEGG pathways for the universal adjuvant, MRh4-679; (**b**) enriched BPs for the universal adjuvant, MRh4-679; (**c**) enriched KEGG pathways for the anticancer adjuvant, SBsib-711; (**d**) enriched BPs for the anticancer adjuvant, SBsib-711; (**e**) enriched KEGG pathways for the antifungal adjuvant, LSsty1-174; (**f**) enriched BPs for the antifungal adjuvant, LSsty1-174.

Adopting systems biology during vaccine development, known as systems vaccinology, is helpful in improving insight into protection and triggered immune responses [38]. KEGG pathways and Gene Ontology (GO) terms can provide a more accurate and clearer panorama for the underlying biological processes of the identified adjuvants. These pathways are applied in omics data, systems biology, and drug development studies [39]. Therefore, using the STITCH database, the KEGG pathways and biological processes affected by each adjuvant were analyzed and are reported in Supplementary Materials S4. In addition, some of the most relevant targets are plotted in Figure 11. It is proposed that the new adjuvant fingerprints should be compared to a benchmark adjuvant [40]. We used an alum adjuvant (aluminum hydroxide) as the reference for comparison. Interestingly, “leukocyte migration” was defined as the main biological process that is affected by the alum derivative with a false discovery rate (FDR) of 0.0268 (FDR < 0.05).

At least 90 KEGG pathways and 409 biological processes can be affected by the universal adjuvant, according to the enrichment analysis report (Supplementary Materials S4). In addition to chemokine signaling and T-cell signaling pathways, MRh4-679 interferes with bacterial (shigellosis, salmonella, and *Vibrio cholerae*) and viral (hepatitis C, hepatitis B, HTLV-1, and influenza A) infections. The most significant affected pathway is interference with proteoglycans in cancer (Figure 11a). Proteoglycans are involved in cancer angiogenesis [41], which aligns with the antiangiogenic characteristic calculated by the machine learning approach in this study. The universal adjuvant affects various pathways, including focal adhesion, Rap1, Ras, PI3K/AKT, and mTOR signaling [42,43]. Prostate, pancreatic, and bladder cancers are targeted significantly. Toxoplasmosis, tuberculosis, malaria, and leishmaniasis are also targeted by MRh4-679, with a lower probability but still in the acceptable statistical range. Interestingly, the biological processes affected by the universal adjuvant are both the TLR4 signaling pathway and the MyD88-independent TLR signaling pathway (Figure 11b).

The anticancer adjuvant (SBsib-711) targets the molecular players of cell adhesion and migration, which are critical for cancer metastasis, such as focal adhesion (FA) and extracellular matrix (ECM) receptors (Figure 11c). FAs are protein complexes attaching cells to the ECM cytoskeleton, providing actin–integrin links [44]. Anticancer peptides affect cell-adhesion molecules (CAMs) to prevent tumor metastasis [45]. The KEGG pathway analysis shows that the anticancer adjuvant targets glioma (Supplementary Materials S4), which is achievable because the ACP candidate is predicted to pass the BBB. Peptidyl-glutamic acid carboxylation is the top biological process affected by the anticancer adjuvant. Peptidyl-glutamic acid carboxylation is involved in the carboxylation process of TAM receptor ligands [46]. TAM (Tyro3, Axl, and MerTK) is homologous to tyrosine kinase receptors and is involved in tumorgenicity and innate immunity regulation. TAM inhibition is a novel target in checkpoint blockade therapy [47]. In addition, the anticancer adjuvant biological processes include regulating leukocyte chemotaxis and immune system processes (Figure 11d).

According to the pathway enrichment analysis, the antifungal adjuvant (LSsty1-174) is predicted to target TLR and cytokine-mediated signaling pathways (Figure 11e,f). However, genes involved in allograft rejection, graft-versus-host disease, autoimmune thyroid disease, rheumatoid arthritis, and inflammatory bowel disease are affected by the antifungal adjuvant (Supplementary Materials S4). This effect can be due to the candidate’s strong induction of the immune system, which is valuable information regarding reactogenicity and should be monitored carefully through in vivo analyses.

Although the antitubercular adjuvant showed interference with tuberculosis according to the KEGG pathway and was involved in chemotaxis and innate immune responses according to the enriched biological processes, the off-target effects, especially on the neural system, should be monitored during in vivo studies (Supplementary Materials S4). Unfortunately, submitting the antiviral peptide to the STITCH database did not provide us with a structure with chemical similarity according to the Tanimoto score. Hence, the immune profiling of this candidate could not be further validated using systems biology.

## 3. Discussion

Except for live-attenuated vaccines, regardless of the vaccine type and delivery system, adjuvants are required in almost all vaccine platforms [9]. With a low potential to trigger autoimmune responses, subunit vaccines are considered safe with few side effects, and their production is economically feasible. However, they are poorly immunogenic by themselves [12]. Therefore, the incorporation of adjuvants is mandatory for subunit vaccines. The first generation of adjuvants was discovered serendipitously by direct in vivo studies. Then, advances in high throughput screening methods by in vitro measurement of adjuvant-associated cellular responses facilitated adjuvant discovery. Reporter cells that express selected innate immunity receptors or cell lines, like the human monocytic THP-1, are used to screen new adjuvants [48]. However, the bottleneck that delays translating innate immune receptor agonists into adjuvants is that the in vitro and in vivo adjuvanticity results are usually uncorrelated [11]. One of the reasons is that adjuvants do not necessarily affect a single target. On the other hand, TLR induction results in the up- and/or downregulation of a wide range of molecules. Thus, researchers believe that it is critical for adjuvant discovery to move toward multiparameter measurements coupled with machine learning and computational data analyses [49,50]. Although animal investigations are indispensable for defining the utility of adjuvants, they have several limitations. For example, the PRRs of each animal and human species have different specificities, and PRR expression patterns across species vary [51]. Recently, humanizing computational models have been proposed to shed light on these differences [52,53]. This strategy can also result in the accelerated discovery of AMPs as novel antibiotics by artificial intelligence [54,55,56]. Although they are of great interest, the immunomodulatory properties of AMPs have been less considered [57]. Our study was conducted to find bifunctional peptides that modulate the immune system and target pathogens or tumor cells. This goal was achieved by mining short immunoadjuvant fragments encrypted in arthropod HDPs using various computational analyses.

During the natural antimicrobial immune response, the induction of TLRs initiates a strong activation of APCs, leading to the upregulation of cell surface MHCs and costimulatory molecules, along with the production of cytokines and chemokines. Therefore, compounds that bind to TLRs hold high expectations for finding efficient immunoadjuvants. Peptide moieties mainly induce cell surface TLRs, including TLR1, TLR2, TLR4, TLR5, and TLR6, which results in the secretion of cytokines like IL6 and TNF-α and polarization of naïve T-cells into Th1 and Th2 immune activation [58]. Within this study, we have deeply analyzed five novel immunomodulatory peptides with antiviral, antifungal, antitubercular, anticancer, and pan-antimicrobial properties. The WALK244.04 peptide, an experimentally validated AMP, was employed as the positive control for the validation of both the universal adjuvant (MRh4-679) and anticancer (SBsib-711) peptides, as they all complexed with same receptor, TLR4/MD2. WALK244.04 has shown an MIC (minimum inhibitory concentration) value of approximately 4.0–8.0 µg/mL against Gram-positive (*Bacillus subtilis* and *Staphylococcus aureus*) and Gram-negative bacteria (*Escherichia coli* and *Shigella dysentariae*) [31]. The parallel performances of docking and MD simulation studies for WALK244.04, as a confirmed ligand of TLR4, with the identified TLR ligands in this study reconfirm the utility of the novel candidates for further trials. We compare the amount of free energy of binding (ΔG bind) and the number of interactions between ligands and receptors in Table 4. The universal and anticancer peptides that interact with TLR4/MD2 have a stronger binding affinity and a higher number of interactions than the receptor compared to the positive control. Considering the important role of flexibility in protein–peptide interactions, we employed an MD simulation assessment. In the MD study of the docked proteins, it was observed that all protein–peptide complexes exhibited various peaks with a deformability index approximating 1.0. Deformability shows protein flexibility, while the B-factor is associated with protein mobility [32]. The B-factor analysis of the MRh4-679-TLR4/MD2 and SBsib-711-TLR4/MD2 complexes revealed fewer significant hinges than WALK244.04-TLR4/MD2. Furthermore, both aforementioned complexes demonstrated lower eigenvalues than the positive control, showing enhanced stability and flexibility in the molecular motion of the docked complexes [33].

The continuous risk of existing viruses and the emergence of new viral species necessitate ongoing research for novel antiviral compounds and prophylactic vaccination [59]. In this study, the SPalf2-453 peptide (HIRRRPKFRK), derived from a crab, was the most optimal immunomodulatory peptide with antiviral properties. TLR2 detects most types of PAMPs and covers many ligands due to its heterodimerization ability with TLR1 [60]. Viral coat proteins and glycoproteins can be detected by TLR2 or TLR2 heterodimers [61,62]. It is known that TLR2 agonists show a Th2-polarized response [63]. Th1 cytokines include IFN-γ, TNF-α, and IL-2, whereas Th2 cytokines are IL-4, IL-5, IL-10, and IL-13. IL-6 defines the Th1/Th2 ratio, which inhibits Th1 differentiation and promotes Th2 induction [64]. According to our results (Table 2), SPalf2-453 is a mild inducer of IL-2, IFN-γ, IL-4, and IL-13, which might result in balanced rather than biased Th1/Th2-associated responses. Balanced responses are favorable in immune challenges with a wide range of immune protection [65,66]. The MD simulation of the TLR1/TLR2-SPalf2-453 complex indicated minimal deformation points at the beginning and end of the complex, with deformation scores higher than 0.6. This indicates structural stability and low susceptibility to deformation. Covariance mapping and elastic network analysis also confirm the strong structural correlation of the complex. As observed in Table 2, SPalf2-453 is a highly cationic peptide with cell penetration potency. Cell-penetrating peptides (CPPs) are carriers for delivering molecules through the impermeable membrane barrier [67]. Although these carriers mainly transport macromolecular species such as proteins [68,69], some CPPs show intrinsic biological characteristics like antiviral activity [70]. For example, LL-37 is an arginine-rich immunomodulatory CPP [71]. Because viruses are obligatory intracellular parasites, antiviral peptides classified as CPPs are valuable in eradicating viral infections [72]. Conjugation of CPPs to antigens in vaccine formulations increases the vaccine’s potency, elevates the accumulation of antigens in draining lymph nodes, and results in T-cell priming and expansion [73]. SPalf2-453 showed potential to pass the blood–brain barrier (BBB) and is applicable to target viral encephalitis.

About 90% of infected patients with *M. tuberculosis* suffer from the so-called long-term stand-off state, as the pathogen largely survives inside the host cells. Only 10% of infected patients display active tuberculosis [74]. Therefore, there is a clear rationale for designing in cellulo therapeutics to target intracellular *Mycobacterium*, the cause of latent tuberculosis [75]. Cell wall fragments of bacterial species such as *Mycobacterium* can be sensed by TLR2 [76]. TLR2 mediates the macrophage and mycobacteria interaction and activates macrophages to eradicate intracellular parasites [77]. All observed interactions in the TLR2-PPpp113-266 complex binding site align with previous reports [78]. The wasp-derived antitubercular candidate in this study induces the production of IL-2 and TNF-α, as well as IL-4 and IL-10; hence, a potentially balanced Th1/Th2 ratio is expected. PPpp113-266, the immunomodulator peptide candidate with antitubercular properties, was shown to be a CPP, which is promising for the eradication of intracellular *Mycobacteria*.

LSsty1-174, an optimal antifungal peptide with immunomodulatory characteristics, is rich in cysteine residues. Because of the presence of three cysteine residues, intramolecular disulfide bonds can form within this peptide. There are various examples of cysteine-rich peptides with antifungal activity [79]. Human β-defensins and plant-derived antifungal peptides are examples of potent compounds against fungal pathogens such as *Candida albicans* [80]. LSsty1-174 induces IL-2, TNF-α, IL-4, IL-10, and IL-6 which seems to be a Th1/Th2-mixed response.

Immunomodulators can dominate immunotolerance in the TME by evoking antitumor immune responses. By repressing Treg cell activity and CTL expansion, TLR agonists have shown tumor growth suppression [24]. Current immunoadjuvants weakly trigger cellular Th1 and CD8^+^ T-cell responses, which are required to moderate antitumor immunity [81]. Therefore, novel strategies to elevate the effect of anticancer agents by triggering immune responses against tumors and for addition in cancer immunotherapy are highly required. TLR4 ligands are valuable targets for this purpose [82]. The identified universal and anticancer immunomodulatory peptides in this study were the main ligands of the TLR4/MD2 complex. Immunomodulatory peptides interacting with TLR4 activate NF-κB through the MyD88 pathway, resulting in the secretion of pro-inflammatory cytokines and polarization toward Th1 responses. Notably, through toll/interleukin-1 receptor domain-containing adapter inducing interferon-β (TRIF) pathway, TLR4 ligands can produce a low level of type I interferons [83]. SBsib-711, the most optimal ACP, is a lysine-rich cationic peptide with a charge of +5. As tumor cells are highly anionic, targeting cationic ACPs specifically for tumor cells is feasible [84]. SBsib-711 has an isoelectric point (pI = 11.34) higher than physiological pH, which leads to a total positive net charge with high potency for accumulation in the acidic tumor microenvironment (TME). According to the prediction of the INSP (Identification Nucleus Signal Peptide) [85], SBsib-711 potentially targets the nucleus. ACPs that can localize inside the nucleus might exert their anticancer effect by deterring DNA synthesis or interfering with proteins involved in cell division [86]. PD-1 and PD-L1 are on T-cells and tumor cells, respectively. PD-1/PD-L1 interaction inhibits CTL, resulting in cancerous cell escape from the host immune surveillance. Neo-adjuvants that block such T-cell checkpoints are promising anticancer agents [87]. SBsib-711 binds PDL-1 and blocks its interaction with PD-1. In addition to adaptive immune checkpoints, innate immune checkpoints such as macrophages have been of great interest recently. CD47, a “do not eat me” signal, prevents tumor phagocytosis by macrophages [88]. SBsib-711 binds CD47 and inhibits the antiphagocytic action of CD47. Finally, SBsib-711 putatively indices IL-2 production and is preferred to recombinant IL-2 in cancer immunotherapy [89].

In addition to erythema, swelling, nodule formation, and abscess in the injection site, the first generation of adjuvants, such as alum salts and emulsions, only recruit a small population of the immune cells and induce a Th2-dominated immune response [90]. On the other hand, LPS-derived adjuvants mainly trigger a Th1-biased response. Th1-mediated responses are preferred for viral pathogens, and Th2-dominated responses are suitable for antiparasitic immune responses. For example, alum is a poor adjuvant for influenza, malaria, or tuberculosis vaccines [29]. Therefore, the wide breadth of the immune response and a Th1/Th2-balanced response are preferred for discovering universal vaccines [91]. MRh4-679, a cationic arginine-rich shrimp-derived peptide, was the most potent ligand for TLR4-MD2 and was defined as the universal adjuvant. TLR4 ligands are putatively safe, efficacious, and universal [81,82]. Although a Th1-polarized response is expected, IL-2 and IL-13 were induced similarly in favor of a balanced associated response. A broad immune response is expected as it showed parallel biological functions. Therefore, it can act putatively as a pan-antimicrobial and universal immunoadjuvant. For example, according to the CAMPR3 program, it was predicted to be antimicrobial, and according to the dbAASP program, it shows activity with an MIC lower than 25 μg/mL against *Pseudomonas aeruginosa* and *Escherichia coli*. MRh4-679 is predicted to be a CPP with antitubercular and antiviral properties. It has antiangiogenic properties, and the anticancer effect can be due to the inhibition of angiogenesis [92].

Despite all of the advances, machine learning-based data still have some limitations. Prediction by machine learning requires input datasets, defined features, and appropriate algorithms. Suppose positive training datasets are composed of a high number of synthetic peptides. In that case, data deviate from natural wisdom to artificial peptides, which is undesirable when the goal is proteome or peptidome scanning [93]. Because negative results are rarely published, the preparation of negative training datasets is difficult and may result in a prediction biased toward large datasets [94]. Selecting the features is also crucial. Programs that are developed using a high number of redundant, colinear, and highly correlated features without considering causality deal with overfitting, which is an undesirable machine learning behavior [95]. Although it is proposed to select a machine learning predictor based on the performance, such as sensitivity, specificity, and accuracy [93], further experimental approval for each optimal candidate is required. For example, IC_50_ (50% inhibitory concentration) or MIC values parallel to safety tests should be determined for clinically resistant pathogens [96]. In addition to in vitro studies, in vivo administration of the identified candidates alone or combined with other antibiotics or AMPs is mandatory. For example, it has been shown that at least two β-defensins are required to restrict bacterial growth in the trachea [97].

Within this study, we proposed a “systems biology” approach, which can be specifically annotated “systems adjuvantology”, to define the fingerprints or immunological signatures of peptides. This strategy provides a holistic view for further in vitro and in vivo immunogenicity and reactogenicity measurements. Analysis of the biological processes and KEEG pathways showed that various but overlapping patterns would be activated upon adjuvant administration. The most consistent data were obtained for the universal and anticancer immunoadjuvants, supporting the machine learning- and docking-retrieved results. Both universal and anticancer adjuvants affect immune-response-regulating pathways and cancer progression pathways. In addition, the universal adjuvant was involved in the defense responses against a broad spectrum of microbes (Supplementary Materials S4). This finding aligns with the complexity of innate immunity signals and effectors. Although the antifungal adjuvant targeted TLR, autoimmune-triggered side effects should be defined in further experiments, especially in immunocompromised patients [98]. In addition, Alum adjuvant, LL-37, a C-terminal extension of human cathelicidin, as a well-known immunomodulator and AMP, was analyzed using the same approach. Chemokine, TLR, TNF, NOD-like receptor, and NF-kb were some of the signaling pathways identified according to the KEGG pathway analysis of LL-37 (Supplementary Materials S4), which agrees with experimental reports [99,100,101,102]. More than 500 enriched biological responses that dominantly involve immune system processes are identified to be affected by LL-37 according to our analyses (Supplementary Materials S4), wherein leukocyte migration and inflammatory responses are the top pathways.

In line with other studies, our investigation showed that decoding the genome, peptidome, and proteome results in identifying functional domains, motifs, or encrypted fragments with standalone biological activity [103]. For example, molecular de-extinction of the paleo-proteome of extinct organisms is an intriguing approach to mining and expanding AMPs [104]. The docking analysis and systems biology combination can be applied to validate the first steps of vaccine development [105]. It is proposed to pair each adjuvant with the corresponding antigen to define adjuvanticity in vivo markers and perform immune profiling to select the optimal adjuvant for each antigen in the vaccine [40].

## 4. Materials and Methods

### 4.1. Identification of Immunomodulatory Peptides and Their Biological Functions

Out of more than 700 collected invertebrate AMPs, 643 validated AMPs from arthropods were retrieved from the InverPep database [30] (http://ciencias.medellin.unal.edu.co/gruposdeinvestigacion/prospeccionydisenobiomoleculas/InverPep/public/home_en) (accessed on 26 January 2024). These AMP data in IverPep were collected from literature surveys and AMP databases. The peptide list in this database is classified into nine phyla of invertebrates, including Annelida, Arthropoda, Echinodermata, Mollusca, Nematoda, Chordata, Platyhelminthes, Placozoa, and Cnidaria. We filtered the list restricted to Arthropoda to collect full-length AMP sequences. These AMPs were used as the parent sequence to mine cryptic short immunomodulatory peptides. The Raghava research group developed the VaxinPAD program, an SVM-based hybrid model, using machine learning methods with an accuracy of 95% for identifying immunomodulatory peptides. The identified candidates are immunostimulants called A-cell epitopes due to the stimulation of APCs [106]. The FASTA format of the AMPs collected from the InverPep database was submitted to the “protein-based vaccine adjuvants” panel of the VaxinPAD program using default settings, such as the dipeptide composition method. The fragment length was set once to 10 and another time to 15 residues (https://webs.iiitd.edu.in/raghava/vaxinpad/protein.php) (accessed on 26 January 2024). This approach extracted immunomodulatory peptides with a length of 10 and 15 amino acid residues, encrypted in AMPs with the putative ability to induce TLRs on APCs. Extracted 10-mer and 15-mer peptides from each species with an SVM score ≥ 0.7 were kept for further efficacy and safety analyses. To annotate the retrieved immunomodulatory peptides, the first letter of the genus and species were indicated with capitals, followed by the three first letters of the parent AMP and the code number in the InverPep database. For example, the immunomodulatory peptide, derived from code 3 of the arthropod phylum with the scientific name *Acalolepta luxuriosa* and parent AMP name “defensin 1 precursor” was annotated as “ALdef1-3”. Allergenicity, hemotoxicity, and cytotoxicity are safety criteria that should be ruled out for effective peptides. Therefore, nonallergic, nonhemolytic and nontoxic immunomodulatory peptides were identified using AllerTOP v.2, HemoPI, and ToxinPred programs [107,108,109]. The AllerTOP v.2 program, with an accuracy of approximately 85.3%, applies the kNN method for prediction and reports the output as probable allergen or nonallergen. HemoPI, which predicts RBC hemolysis, is an SVM-based machine learning program with an accuracy of approximately 95%. Scores higher than 0.5 are considered hemolytic, compared to melittin, with a score of 0.8 as the positive control. ToxinPred is an SVM-based machine learning approach to predicting the toxicity of peptides, with an accuracy of 94.5%. Using batch submission and according to the default settings, the FASTA format of 10-mer and 15-mer immunomodulatory peptides was submitted to be classified as toxic or nontoxic. The physiochemical properties of peptides and their cell-penetration ability were collected from the VaxinPAD, dbAASP, and MLCPP programs [110,111]. In parallel to the APC induction score, VaxinPAD reports the physiochemical properties, such as hydrophobicity, hydropathicity, charge, and isoelectric point, of peptides. The propensity to in vitro aggregation, an undesirable physiochemical property for peptides, was calculated using the Moon and Fleming scale of the dbAASP. The dbAASP database has a “property calculation mode” under the Tools menu bar. MLCPP is a machine learning predictor composed of two layers. In the first layer, it predicts whether a peptide has cell-penetration ability with an accuracy of 89%. The second layer is performed with a random forest algorithm and predicts whether a CPP has low or high uptake efficiency with an accuracy of 72%. The BBB permeation was defined by the “predict” mode of the B3Pred program [112]. B3Pred is a random-forest-based tool with an accuracy of 85.08% for predicting blood–brain barrier-penetrating peptides. Cytokine-inducing peptides were identified using IL2Pred, IL4pred, IL-6Pred, IL-10Pred, TNFepitope, and IFNepitope, as mentioned earlier [27]. IL2Pred is a random-forest-based machine learning program with an accuracy of 73.25% for predicting interleukin-2-inducing peptides. Interleukin-4-inducing peptides were identified using the default settings under the virtual screening mode of the IL4pred program. IL4pred is an SVM-based machine learning classifier with an accuracy of 75.76%. Prediction of interleukin-6-inducing peptides was defined using IL-6Pred, using a random-forest-based method with an accuracy of 75.79%. Interleukin-10-inducing peptides were identified by IL-10Pred, an SVM-based model with an accuracy of 78.42%. TNF-α-inducing epitopes were predicted using the TNFepitope webserver that applies hybrid alignment-based and alignment-free methods with an AUROC (area under the ROC curve) of 0.83 for human hosts. IFNepitope was used to determine interferon-gamma-inducing peptides using the hybrid approach (motif+SVM) with an accuracy of 82.10%. In addition, the induction of IL-13 was assessed using the IL13pred program using the eXtreme Gradient Boosting (XGB) probability method, with an accuracy of about 71% [113]. The MetaiAVP, AntiFP, and AntiTbPred programs were used to identify immunoadjuvant peptides with antiviral, antifungal, and antitubercular characteristics, respectively [27,28,29]. MetaiAVP is a metapredictor for large-scale prediction of antiviral peptides with an accuracy of 95.20%. Antifungal peptides were identified using the AntiFP program, an SVM-based model, with an accuracy of 83.33%. With an accuracy of 76.56%, AntiTbPred defines antitubercular peptides using the SVM method. Finally, immunomodulatory candidates predicted to be classified as anticancer peptides (ACPs) were defined using AntiCP 2.0 (an SVM-based model with an accuracy of about 71%) and CancerGram (a random-forest-based model with an accuracy of about 85%) [114,115].

### 4.2. Retrieval of TLR Structures as Receptors

The crystal structures of human TLRs (hTLR) with the ability to interact with peptide ligands were retrieved from the protein databank as follows. TLR1 (PDB ID: 6NIH), TLR2 (PDB ID: 6NIG), TLR4 (PDB ID: 3FXI), and TLR5 (PDB ID: 3J0A). TLR6, which lacked a crystal structure, was modeled with Swiss-Model homology modeling using PDB ID: 3A79.1.B as the template [116,117,118,119]. For some ligands, hTLR2 must bind with TLR1 or TLR6 and form a heterodimer to activate in vivo. Therefore, heterodimers of TLR2/TLR1 and TLR2/TLR6 were also studied as receptors. To obtain the TLR2/TLR6 complex, we employed protein–protein docking via the ClusPro 2.0 program to assemble them into a unified structure as the receptor [120]. The TLR1/TLR2 heterodimer was available in the protein databank (PDB ID: 2Z7X).

### 4.3. Receptor and Ligand Preparation

TLR preparation was conducted using the Chimera program [121]. Native ligands and solvent molecules were removed. Then, polar hydrogen atoms and Kollman charges were added, followed by energy minimization. The receptor binding pocket was determined using the P2Rank program (http://siret.ms.mff.cuni.cz/p2rank) (accessed 26 January 2024) [122] and Molecular Operating Environment (MOE) [123]. The most potent and safe immunoadjuvant peptides, such as the universal adjuvant and immunomodulatory peptides with antiviral, antitubercular, antifungal, and anticancer characteristics, were selected as ligands for docking analysis. Peptide ligands were modeled using the I-TASSER program (https://zhanggroup.org/I-TASSER/) (accessed 26 January 2024), and 3D models with the highest confidence score were selected for energy minimization by 3D Refine, as mentioned earlier [124,125,126,127].

### 4.4. Molecular Docking and Molecular Dynamics Simulation Studies

To find the target TLR for ligands, each ligand was docked with cell surface TLRs, including TLR1, TLR2, TLR4, TLR5, TLR6, TLR1/2, and TLR2/6, using the ClusPro 2.0 tool. This web server performs docking with the PIPER program (https://cluspro.org/login.php) (accessed 26 January 2024), which relies upon a highly efficient fast Fourier transform (FFT) correlation technique [120]. The MOE dock software (version 2022) validated the optimal complex between each ligand and its target receptor [123]. MOE was also used for an in-depth visualization of ligand–receptor interactions. The iMODs program was used to conduct molecular dynamics simulation (MD) studies [128]. This program predicts the collective functional motions of biomacromolecules in internal coordinates (dihedrals) using the normal mode analysis (NMA) method. It provides detailed analyses, including B-factor plots, deformability, eigenvalues, covariance matrix, and elastic network [128]. The docked complex between receptor and ligand was used as an input to calculate the molecular motion and stability, with all parameters set to default.

### 4.5. Identification of In Vivo Target Pathways

We employed a systems biology approach to define the pathways and functions that are affected by the identified immunoadjuvants for in vivo conditions. For that purpose, the peptide sequences of the best immunoadjuvant candidates were converted to the “SMILES” format, a Simplified Molecular-Input Line-Entry System, using BIOPEP-UWM [129]. The converted chemical structure was submitted to the STITCH database [130], a Search Tool for Interaction of Chemicals, to find the target pathways and biological processes affected by each immunoadjuvant. The organism was restricted to Homo sapiens. The settings were adjusted using the minimum required interaction score (0.15) and a maximum number of 50 interactions in the first and second shells. Through the “Analysis” panel, the Gene Ontology (GO) enrichment analysis, including biological processes and KEGG pathways, was acquired. The outputs are sorted according to the FDR by the program. An FDR < 0.05 is considered significant.

## 5. Conclusions

We used machine learning methods to identify immunomodulatory peptides derived from arthropod AMPs. Cytokine induction properties and target microorganisms were defined according to the objectives. This multimechanistic approach is valuable for decreasing microbial resistance against antibiotics and triggering the immune system as vaccine adjuvants. Molecular docking and simulation studies assisted in identifying the target TLR for each candidate compared to the experimentally validated positive control. The arginine-rich universal adjuvant (KPAIRRLARR) and lysine-rich anticancer peptide (KLKRGAKKAL) both interact with the TLR4/MD2. These peptides showed a stronger binding affinity and a higher number of interactions with the receptor compared to the antibacterial positive control. Systems biology investigation reconfirmed the interaction of the identified universal and anticancer adjuvants with microbial or cancer cells and immune system modulation compared to the alum adjuvant and LL-37 peptide as the positive control. The antitubercular peptide (RVQERRFKRI) and antifungal adjuvant (PCVQQPCPKC) strongly interacted with TLR2. Pathway enrichment analysis reconfirmed their involvement in fighting tuberculosis and stimulating the immune system, respectively. However, their potential reactogenicity should be further investigated by experimental analysis. Finally, the placement of such immunomodulators as multifunctional adjuvants is in its infancy, and further trials are required to determine the most optimal production platform, formulation, and delivery approach.

## Figures and Tables

**Table 1:** A collection of 55 arthropods retrieved from the InverPep database [30].

Class	Order	Family	Genus	Species
Arachnida 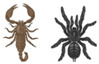	Ixodida	Ixodidae	Ixodes	*Ixodes ricinus*
				*Ixodes scapularis*
				*Ixodes sinensis*
			Rhipicephalus	*Rhipicephalus haemaphysaloides*
				*Rhipicephalus microplus*
			Dermacentor	*Dermacentor silvarum*
	Araneae	Theraphosidae	Cyriopagopus	*Cyriopagopus hainanus*
			Acanthoscurria	*Acanthoscurria gomesiana*
		Oxyopidae	Oxyopes	*Oxyopes takobius*
				*Oxyopes kitabensis*
		Zodariidae	Lachesana	*Lachesana tarabaevi*
		Lycosidae	Hogna	*Hogna carolinensis*
	Scorpiones	Buthidae	Parabuthus	*Parabuthus schlechteri*
			Olivierus	*Olivierus martensii*
			Vaejovis	*Vaejovis punctatus*
			Androctonus	*Androctonus australis*
			Mesobuthus	*Mesobuthus eupeus*
		Scorpionidae	Pandinus	*Pandinus imperator*
Insecta 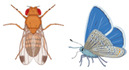	Hymenoptera	Vespidae	Vespa	*Vespa tropica*
			Mischocyttarus	*Mischocyttarus phthisicus*
			Eumenes	*Eumenes magnifica*
		Formicidae	Neoponera	*Neoponera goeldii*
		Pteromalidae	Pteromalus	*Pteromalus puparum*
		Melittidae	Macropis	*Macropis fulvipes*
	Lepidoptera	Saturniidae	Hyalophora	*Hyalophora cecropia*
			Antheraea	*Antheraea pernyi*
		Noctuidae	Chloridea	*Chloridea virescens*
		Psychidae	Oiketicus	*Oiketicus kirbyi*
		Pyralidae	Galleria	*Galleria mellonella*
		Bombycidae	Bombyx	*Bombyx mori*
		Sphingidae	Manduca	*Manduca sexta*
		Erebidae	Hyphantria	*Hyphantria cunea*
	Diptera	Calliphoridae	Calliphora	*Calliphora vicina*
			Lucilia	*Lucilia sericata*
		Tephritidae	Ceratitis	*Ceratitis capitata*
			Bactrocera	*Bactrocera dorsalis*
		Simuliidae	Simulium	*Simulium bannaense*
		Drosophilidae	Drosophila	*Drosophila melanogaster*
	Hemiptera	Cicadidae	Cryptotympana	*Cryptotympana dubia*
			Cicada	*Cicada flammata*
		Pentatomidae	Podisus	*Podisus maculiventris*
	Coleoptera	Cerambycidae	Acalolepta	*Acalolepta luxuriosa*
		Chrysomelidae	Chrysomelinae	*Chrysomelinae atrocyanea*
	Blattodea	Blattidae	Periplaneta	*Periplaneta americana*
	Orthoptera	Acrididae	Locusta	*Locusta migratoria*
Malacostraca 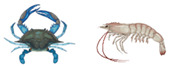	Decapoda	Portunidae	Scylla	*Scylla paramamosain*
				*Scylla serrata*
			Portunus	*Portunus trituberculatus*
			Callinectes	*Callinectes sapidus*
		Penaeidae	Litopenaeus	*Litopenaeus vannamei*
				*Litopenaeus stylirostris*
		Astacidae	Pacifastacus	*Pacifastacus leniusculus*
		Palaemonidae	Macrobrachium	*Macrobrachium rosenbergii*
		Oregoniidae	Hyas	*Hyas araneus*
Merostomata	Xiphosura	Limulidae	Limulus	*Limulus polyphemus*
Chilopoda	Scolopendromorpha	Scolopendridae	Scolopendra	*Scolopendra subspinipes*

**Table 2:** Physicochemical characteristics and cytokine induction of the most optimal immunomodulatory (IM) peptides with defined antimicrobial or anticancer properties.

	Peptide ID	Sequence	pI	Charge	GRAVY	CPP	BBBp	IL-4	IL-10	IL-13	IL-2	IL-6	TNFα	IFN-Ɣ
Universal IM	MRh4-679	KPAIRRLARR	12.48	+5	−1.16	✓	✓	X	X	✓ 0.78	✓ 0.95	✓ 0.35	X	✓
Antiviral IM	SPalf2-453	HIRRRPKFRK	12.49	+6	−2.33	✓	✓	✓ 0.30	X	✓ 0.30	✓ 0.75	✓ 0.25	✓ 0.56	✓
Antifungal IM	LSsty1-174	PCVQQPCPKC	8.26	+1	−0.40	X	✓	✓ 0.28	X	✓ 0.28	✓ 0.85	✓ 0.38	✓ 0.55	X
Antitubercular IM	PPpp113-266	RVQERRFKRI	12.01	+4	−1.74	✓	✓	✓ 1.30	✓ 0.60	✓ 0.06	✓ 0.93	✓ 0.30	✓ 0.58	X
Anticancer IM	SBsib-711	KLKRGAKKAL	11.34	+5	−0.93	✓	✓	X	X	✓ 0.83	✓ 0.87	✓ 0.35	✓ 0.64	X

X: indicates a negative response; ✓: indicates a positive response with scores of cytokine-inducing peptides; pI: isoelectric point; BBBp: blood–brain barrier penetration; CPP: cell-penetrating peptide.

**Table 3:** Interactions between the MRh4-679 ligand (KPAIRRLARR), universal immunoadjuvant, and TLR4/MD2 receptor by defining the type of interaction, distance, and energy of interaction.

Ligand	Receptor Interacting Amino Acids	Type of Interaction	Location of Interaction	Distance (Å)	Binding Energy (kcal/mol)
N 1	LEU 269	H-donor	TLR4 (Chain B)	3.20	−2.1
NZ 7	GLU 266	H-donor	TLR4 (Chain B)	2.94	−5.5
NE 73	SER 120	H-donor	MD-2 (Chain D)	3.38	−0.6
NH2 76	PHE 121	H-donor	MD-2 (Chain D)	3.01	−1.9
NH1 99	ASP 294	H-donor	TLR4 (Chain B)	3.08	−2.4
NH2 100	ASP 294	H-donor	TLR4 (Chain B)	3.29	−3.0
NE 150	GLU 92	H-donor	MD-2 (Chain D)	2.93	−4.7
NH1 152	VAL 93	H-donor	MD-2 (Chain D)	3.23	−1.3
NH2 153	GLU 92	H-donor	MD-2 (Chain D)	2.96	−5.6
NH2 153	VAL 93	H-donor	MD-2 (Chain D)	3.22	−1.8
OXT 180	ARG 264	H-acceptor	TLR4 (Chain B)	3.02	−2.4
OXT 180	LYS 362	H-acceptor	TLR4 (Chain B)	3.25	−2.4
N 1	ASP 294	Ionic	TLR4 (Chain B)	3.39	−2.3
NH1 99	ASP 294	Ionic	TLR4 (Chain B)	3.2	−3.3
NH1 99	ASP 294	Ionic	TLR4 (Chain B)	3.08	−4.0
NH2 100	ASP 294	Ionic	TLR4 (Chain B)	3.26	−3.0
NH2 100	ASP 294	Ionic	TLR4 (Chain B)	3.29	−2.8
NE 150	GLU 92	Ionic	MD-2 (Chain D)	2.93	−4.9
NH2 153	GLU 92	Ionic	MD-2 (Chain D)	2.96	−4.8
NH1 176	ASP 101	Ionic	MD-2 (Chain D)	3.42	−2.2
NH2 177	ASP 101	Ionic	MD-2 (Chain D)	3.50	−1.9
NH2 177	ASP 101	Ionic	MD-2 (Chain D)	3.71	−1.2
OXT 180	ARG 264	Ionic	MD-2 (Chain D)	3.49	−1.9
OXT 180	ARG 264	Ionic	MD-2 (Chain D)	3.02	−4.3

**Table 4:** Interaction of immunomodulatory (IM) peptides with target TLRs, number of interactions in the ligand–receptor binding pocket, and the free energy of binding.

Ligand	Optimal Complex	Number of Interactions	Binding Energy (kcal/mol)
Positive control	TLR4/MD2-WALK244.04	10	−25.2
Universal IM	TLR4/MD2-MRh4-679	24	−70.3
Antiviral IM	TLR1/2-SPalf2-453	20	−72.1
Antifungal IM	TLR2-LSsty1-174	7	−7.6
Antitubercular IM	TLR2-PPpp113-266	16	−49.6
Anticancer IM	TLR4/MD2-SBsib-711	12	−39.9

## Data Availability

The data presented in this study are available upon request from the corresponding author.

## Supplementary Materials

The following supporting information can be downloaded at: https://www.mdpi.com/article/10.3390/ph17020201/s1, Supplementary Materials S1: Immunomodulatory (IM) peptides applicable as immunoadjuvants with a length of 10 and 15 amino acid residues encrypted in various arthropod AMPs. IM peptides with a score ≥ 0.7 were retrieved using the VaxinPAD program; Supplementary Materials S2: Physiochemical properties and predicted biological functions for potent immunoadjuvants derived from selected arthropods; Supplementary Materials S3: MD simulation studies for the most potent antiviral, antitubercular, antifungal, and anticancer immunoadjuvant peptides; Supplementary Materials S4: KEGG pathway enrichment analysis and biological process (BP) terms for the identified adjuvants. ACP: anticancer; AFP: antifungal peptide; ATB: antitubercular peptide.
