# Supplementary material for: Immunomodulatory Peptides as Vaccine Adjuvants and Antimicrobial Agents
Source: Pharmaceuticals (Basel). 2024 Feb 2;17(2):201. doi: 10.3390/ph17020201 (PMC10892805; doi:10.3390/ph17020201)
Supplement: Supplementary file 1 [file pharmaceuticals-17-00201-s001.zip › Supplementary material S1.pdf]

Supplementary Material S1 Immunomodulatory (IM) peptides applicable as immunoadjuvants with a length of 10 and 15 amino acid residues encrypted in various arthropod AMPs. IM peptides with a score ≥ 0.7 were retrieved using the VaxinPAD program.

**SPECIE**	**Name of parent peptide**	**Antimicrobial peptide**	**Peptide ID**	**Proposed IM sequence from Vaxinpad**
Hyas araneus	Arasin 2	SRWPSPGRPRPFPGRPNPIFRPRPCICVRQPCPCDTY	HAara2-163	PCICVRQPCP
Portunus trituberculatus	PtesALF1	YEALVTSILGKLTGLWHNDSVDFMGHICYFRRRPKIRRFKLYHEGKFWCPGWAPFEGRCKYCVVF	PTesalf1-457	ICYFRRRPKIRRFKL
Macrobrachium rosenbergii	MrH4	MTGRGKGGKGLGKGGAKRHRKVLRDNIQGITKPAIRRLARRGGVKRISGLIYEETRGVLKVFLENVIRDAVTYTEHAKRKTVTAMDVVYALKRQGRTLYGFGG	MRh4-679	KPAIRRLARR
Scylla paramamosain	Arasin-likeSp	SPRVSRRYGRPFGGRPFVGGQFGGRPGCVCIRSPCPCANYG	SPAara-291	CVCIRSPCPC
Scylla paramamosain	Sp-ALF1	YETLIASVLGKLTGLWHNNSVDFMGHTCHFRRRPKVRKFKLYHEGKFWCPGWAPFEGRSRTKSRSGSSREAIKDFVRKALQNGLITQQDATVWVN	SPalf1-452	RRRPKVRKFK
Calliphora vicina	Alloferon	HGVSGHGQHGVHG	CVall-91	SGHGQHGVHG
Limulus	LALF	DGIWTQLIFTLVKNLATLWQSGDFQFLDHECHYRIKPTFRRLKWKYKGKFWCPSWTSITGRATKSSRSGAVEHSVRNFVGQAKSSGLITQRQAEQFISQYN	LPlal-	YRIKPTFRRLKWK

polyphemus			455	YK
Pachycondyla goeldii	Ponericin W3	GIWGTLAKIGIKAVPRVISMLKKKKQ	PGponW3-227	VPRVISMLKK
Pteromalus puparum	PP113	GKWGWIYITILFADVGGFKSSRHPEERRVQERRFKRITRGPD	PPpp113-266	RVQERRFKRI
Ixodes ricinus	DefMT5	GFFCPYNGYCDRCRKKLRRRGGYCGGRWKLTCICIMN	IRdefmt5-704	RCRKKLRRRG
Lachesana tarabaevi	Latarcin-1	SMWSGMWRRKLKKLRNALKKKLKGE	LTlat1-381	GMWRRKLKKL
Mesobuthus martensii	BmKbpp	FRFGSFLKKVWKSKLAKKLRSKGKQLLKDYANKVLNGPEEEAAAPAE	MMbmk-503	SFLKKVWKSKLAKKL
Gastrophysa atrocyanea	Diapause-specific peptide	AVRIGPCDQVCPRIVPERHECCRAHGRSGYAYCSGGGMYCN	GAdia-145	VRIGPCDQVCPRIVP
Lachesana tarabaevi	Latarcin-5	GFFGKMKEYFKKFGASFKRRFANLKKRL	LTlat5-387	FKRRFANLKK
Oiketicus kirbyi	Cecropin	WKPFKKIEKAVRRVRDGVAKAGPAVAVVGQAT	OKcec-603	WKPFKKIEKAVRRVR
Lucilia sericata	LSerStomox1	AGFRKRFNKLVKKVKHTIKETANVSKDVAIVAGSGVAVGAAMG	LSersto1-548	RKRFNKLVKKVKHTI
Galleria	AGm1	ENFFKEKERKGQRIRDAIISRRPRVETLAQAQKIIKGGD	GMagm	RIRDAIIS

mellonella			1-684	RR
Callinectes sapidus	Callinectin	WNSNRRFRVGRPPVVGRPGCVCFRAPCPCSNY	CScal-89	CVCFRAPCPC
Rhipicephalus haemaphysaloides	Rhamp	ERILDLRKTKKSCKNGEVLGCVSGHGPPGCSENECGMGPRPKACFFDCHYGCWCTGKLYRRKRDRKCVPKHECLL	RHrha-419	RRKRDRKCVP
Parabuthus schlechteri	Parabutoporin	FKLGSFLKKAWKSKLAKKLRAKGKEMLKDYAKGLLEGGSEEVPGQ	PSpar-351	SFLKKAWKSKLAKKL
Scylla paramamosain	Sp-ALF2	YEALVASILGKLSGLWHSDTVDFMGHTCHIRRRPKFRKFKLYHEGKFWCPGWTHLEGNSRTKSRSGSARDAIKDFVYKALQNKLITENNAAAWLK	SPalf2-453	HIRRRPKFRK
Scylla paramamosain	Sp-ALF3	YEALVASILGKLSGLWHSDTVDFMGHTCHIRRRPKFRKFKLYHEGKFWCPGWTHLEGNSRTKSRSGSARDAIKDFVYKALQNKLITENNAAAWLK	SPalf2-453-	TCHIRRRPKFRKFKL
Ixodes scapularis	Scapularisin-3	AFGCPFDQGTCHSHCRSIRRRGRRCASFAKRTCTCYQK	ISsca3-531	IRRRGRRCASFAKRT
Macropis fulvipes	Macropin 1	GFGMALKLLKKVL	MFmac1-176	GMALKLLKKV
Vaejovis punctatus	VpAmp1.0	LPFFLLSLIPSAISAIKKI	VPamp1-709	IPSAISAIKK
Drosophila melanogaster	Cecropin-C	GWLKKLGKRIERIGQHTRDATIQGLGIAQQAANVAATARG	DMcecC-120	LKKLGKRIERIGQHT
Androctonus	Androctoni	RSVCRQIKICRRRGGCYYKCTNRPY	AAand-	RQIKICR

australis	n		324	RRG
Cryptotympana dubia	Cryptonin	GLLNGLALRLGKRALKKIIKRLCR	CDcry106	RALKKIIKRL
Bombyx mori	Moricin 2	AKIPIKAIKTVGKAVGKGLRAINIASTANDVFNFLKPKKRKH	BMmor2-64	NFLKPKKRKH
Scylla serrata	SsALF	QYEALVASILGKLSGLWHSDTVDFMGHTCHIRRKPKFRKFKLYHEGKFWCPGWTHLEGNSRTKSRSGSTREATKDFVHKALQNKLITKNSADAWLKG	Ssalf-495	TCHIRRKPKFRKFKL
Litopenaeus vannamei	Histone H2A	SGRGKGGKVKGKSKSRSSRAGLQFPVGRIHRLLRKGNYAERVGAGAPVYLAAVMEYLAAEVLELAGNAARDNKKTRIVPRHLQLAIRNDEELNKLLSGVTIAQGGVLPNIQAVLLPKKTEKK	LVhis-638	KKTRIVPRHLQLAIR
Dermacentor silvarum	Ds-defensin	VPAESEAAHLRVRRGFGCPLNQGACHNHCRSIRRRGGYCSGIIKQTCTCYRN	DSdef-706	LRVRRGFGCP
Portunus trituberculatus	PtALF7	QYEALTAAILTKLSKMWHSDTLNFLGHTCHVSRTPTVKRFKLYWKGKFWCPGWAPFSGTSRTKSRSGSAREATKSFVDQALQRRLITQQEADLWLKG	PTalf7-456	VKRFKLYWKG
Scolopendra mutilans	Scolopin-1	FLPKMSTKLRVPYRRGTKDYH	SMscol-594	TKLRVPYRRG
Periplaneta americana	ISGCock_Contig13_4610?1	YPCKLNLKLGKVPFHF	PAisg13-726	YPCKLNLKLGKVPFH
Galleria mellonella	Gm1	ENFFKEIERAGQRIRDAIISAAPAVETLAQAQKIIKGGD	GMgm1-136	QRIRDAIISA
Acalolepta	Luxuriosin	SVRTQDNAVNRQIFGSNGPYRDFQLSDCYLPLETNPYCNEWQFAYHWNNALMDCERAIYHGCNRTRNNFITLTACKNQ	AL1	RAIYHG

luxuriosa		AGPICNRRRH	ux-2	CNRT
Bombyx mori	Moricin	AKIPIKAIKTVGKAVGKGLRAINIASTANDVFNFLKPKKRKA	BMmor-72	FNFLKPKKRK
Vespa tropica	Mastoparan-VT3	INLKAITALAKKLL	VTmas3-505	LKAITALAKK
Bombyx mori	CECD_BOMMO Cecropin-D precursor	GNFFKDLEKMGQRVRDAVISAAPAVDTLAKAKALGQ	BMcec-70	QRVRDAVISA
Acanthoscurria gomesiana	Gomesin	QCRRLCYKQRCVTYCRGR	AGgom-328	RRLCYKQRCV
Mischocyttarus phthisicus	Mp_mastoparan MP	INWLKLGKKMMSAL	MPmas-180	LKLGKKMMSA
Rhipicephalus microplus	Microplusin	HHQELCTKGDDALVTELECIRLRISPETNAAFDNAVQQLNCLNRACAYRKMCATNNLEQAMSVYFTNEQIKEIHDAATACDPEAHHEHDH	RMmic-375	ECIRLRISPE
Mesobuthus eupeus	Meucin-25	VKLIQIRIWIQYVTVLQMFSMKTKQ	MEmeu25-416	VKLIQIRIWI
Drosophila melanogaster	Drosomycin-2	DCLSGKYKGPCAVWDNEMCRRICKEEGHISGHCSPSLKCWCEGC	DMdro2-110	PCAVWDNEMCRRICK
Oxyopes	Oxyopinin-2	GKFSVFGKILRSIAKVFKGVGKVRKQFKTASDLDKNQ	OKoxy2-	AKVFKGVGKVRK

kitabensis			371	QFK
Pacifastacus leniusculus	Astacidin 1	FKVQNQHGQVVKIFHH	Plast-417	FKVQNQHGQVVKIFH
Oxyopes takobius	Oxt 4a	GIRCPKSWKCKAFKQRVLKRLLAMLRQHAF	OToxt4a-429	RVLKRLLAML
Lachesana tarabaevi	Latarcin-2a	GLFGKLIKKFGRKAISYAVKKARGKH	LTlat2a-382	IKKFGRKAISYAVKK
Scolopendra subspinipes mutilans	Scolopendin 1	MDSFQKIEKIGEGTYGVVYKAKDKVSGRLVALKKIRLENESEGVPSTA	SSMsco1-529	RLVALKKIRL
Macropis fulvipes	Macropin 2	GTGLPMSERRKIMLMMR	MFmac2-177	ERRKIMLMMR
Ceratitis capitata	Ceratotoxin C	SLGGVISGAKKVAKVAIPIGKAVLPVVAKLVG	CCcerC-94	VISGAKKVAKVAIPI
Podisus maculiventris	Thanatin	GSKKPVPIIYCNRRTGKCQRM	PMtha-315	KKPVPIIYCNRRTGK
Lachesana tarabaevi	Cytoinsectotoxin 1-13	GFFGNTWKKIKGKADKIMLKKAVKIMVKKEGISKEEAQAKVDAMSKKQIRLYLLKHYGKKLFKKRPKNCDQ	LTcyt1-13-628	LKHYGKKLFK
Bactro	Bactrocerin	VGKTWIKVIRGIGKSKIKWQ	BDb	IKVIRGI

cera dorsalis	-1		ac1-56	GKS
Bactrocera dorsalis	Bactrocerin-1	VGKTWIKVIRGIGKSKIKWQ	BDbac1-56-	WIKVIRGIGKSKIKW
Vespa magnifica	Vespid chemotactic peptide 5g	FLIIRRPIVLGLL	VMves-579	FLIIRRPIVL
Simulium bannaense	SibaCec	GKLTKDKLKRGAKKALNVASKVAPIVAAGASIAR	SBsib-711	KLKRGAKKAL
Ixodes sinensis	Ixosin-B	QLKVDLWGTRSGIQPEQHSSGKSDVRRWRSRY	ISixoB-169	KSDVRRWRSR
Hyalophora cecropia	Cecropin-A	KWKLFKKIEKVGQNIRDGIIKAGPAVAVVGQATQIAK	HCcecA-322	WKLFKKIEKVGQNIR
Haplopelma hainanum	Oh-defensin	MLCKLSMFGAVLGVPACAIDCLPMGKTGGSCEGGVCGCRKLTFKILWDKKFG	5HHoh-d-431	GCRKLTFKIL
Periplaneta americana	ISGCock_Contig12_4176	VGRKHSILNCIPYLKKKKIMRL	PAisg12-732	LNCIPYLKKKKIMRL
Bombyx mori	Attacin	QAGSFTVNSDGTSGAALKVPLTGNDKNVLSAIGSADFNDRHKLSAASAGLALDNVNGHGLSLTGTRIPGFGEQLGVAGKVNLFHNNNHDLSAKAFAIRNSPSAIPNAPNFNTLGGGVDYMFKQKVGASLSAAHSDVINRNDYSAGGKLNLFRSPSSSLDFNAGFKKFDTPFYRSSWEPNVGFSFSKFF	BMatt-73	SAKAFAIRNSPSAIP
Litopenaeus stylirostris	Ls-Stylicin1	SSFSPPRGPPGWGPPCVQQPCPKCPYDDYKCPTCDKFPECEECPHISIGCECGYFSCECPKPVCEPCESPIAELIKKGGYKG	LSsty1-174	PCVQQPCPKC

Hogna carolinensis	Lycotoxin-2	KIKWFKTMKSIAKFIAKEQMKKHLGGE	HClyc2-363	KIKWFKTMKS
Galleria mellonella	G. mellonella moricin-like peptide A	KVNANAIKKGGKAIGKGFKVISAASTAHDVYEHIKNRRH	GMg.meA-137	AIGKGFKVIS
Cicada flammata	Cicadin	NEYHGFVDKANNENKRKKQQGRDDFVVKPNNFANRRRKDDYNENYYDDVDAADVV	CFcic-619	NFANRRRKDD
Antheraea pernyi	pro-ApCec	APEPKWKFFKKIERVGQNIRDGIIKAGPAVAVVGQATNIAKG	APpro-763	WKFFKKIERVGQNIR
Drosophila melanogaster	CEC3_DROVI Cecropin-3 precursor	GWLKKIGKKIERIGQHTRDATIQGVGIAQQAANVAATAR	DMcec3-121	LKKIGKKIERIGQHT
Heliothis virescens	Cecropin-B	KWKVFKKIEKVGRNIRDGIVKAGPAIAVLGQAN	HVcecB-155	KWKVFKKIEK
Lachesana tarabaevi	Cytoinsectotoxin-1a	GFFGNTWKKIKGKADKIMLKKAVKIMVKKEGISKEEAQAKVDAMSKKQIRLYLLKYYGKKALQKASEKL	LTcyt1a-467	MLKKAVKIMV
Locusta migratoria	Locustin	ATTGCSCPQCIIFDPICASSYKNGRRGFSSGCHMRCYNRCHGTDYFQISKGSKCI	LMloc-179	HMRCYNRCHG
Manduca sexta	Bactericidin B-3	WNPFKELERAGQRVRDAIISAGPAVATVGQAAAIARG	MSbacB3-312	QRVRDAIISA
Hyphantria cunea	Hyphancin IIIE	RWKFFKKIERVGQNVRDGLIKAGPAIQVLGAAKAL	HChypE-166	WKFFKKIERVGQNVR

Pandinus imperator	Pandinin-1	GKVWDWIKSAAKKIWSSEPVSQLKGQVLNAAKNYVAEKIGATPT	PIpan1-365	KVWDWIKSAAKKIWS
Oxyopes kitabensis	Oxyopinin-2d	GKFSVFSKILRSIAKVFKGVGKVRKGFKTASDLDKNQ	OKoxy2b-374	AKVFKGVGKVRKGFK
