# Supplementary material for: Immunomodulatory Peptides as Vaccine Adjuvants and Antimicrobial Agents
Source: Pharmaceuticals (Basel). 2024 Feb 2;17(2):201. doi: 10.3390/ph17020201 (PMC10892805; doi:10.3390/ph17020201)
Supplement: Supplementary file 1 [file pharmaceuticals-17-00201-s001.zip › Supplementary material S3.pdf]

Supplementary Material S3 MD simulation studies for the most potent antiviral, antitubercular, antifungal, and anticancer immunoadjuvant peptides

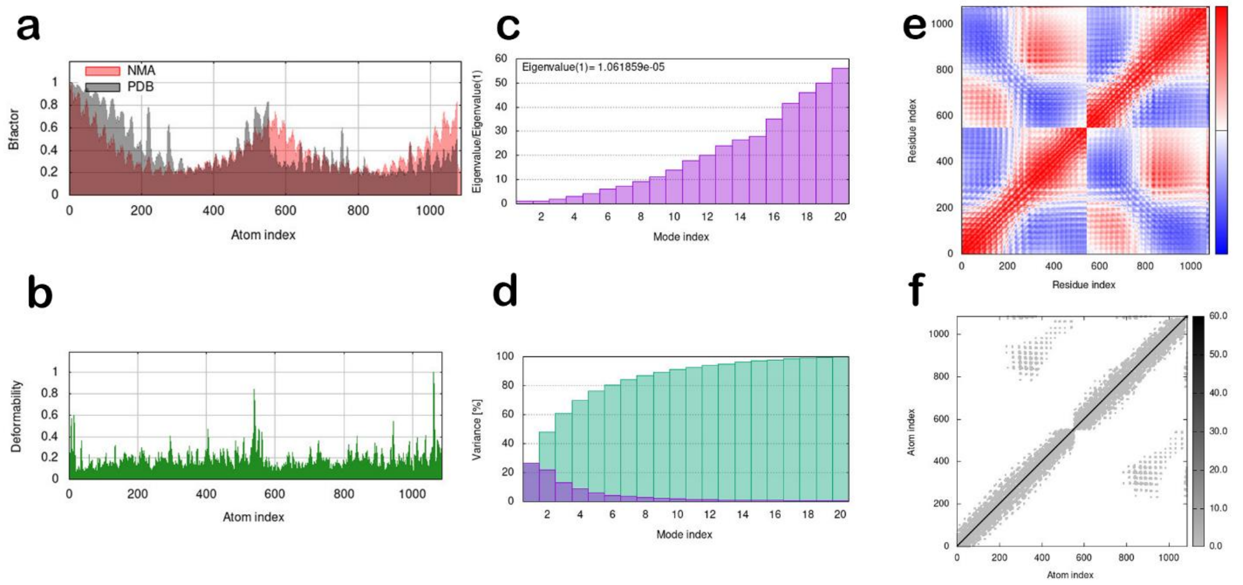

Molecular dynamics simulation of TLR1/TLR2-SPalf2-453 complex as the antiviral adjuvant. (a) B-factor, (b) Deformability, (c) Eigenvalues, (d) Variance, (e) Covariance matrix, (f) The elastic network map.

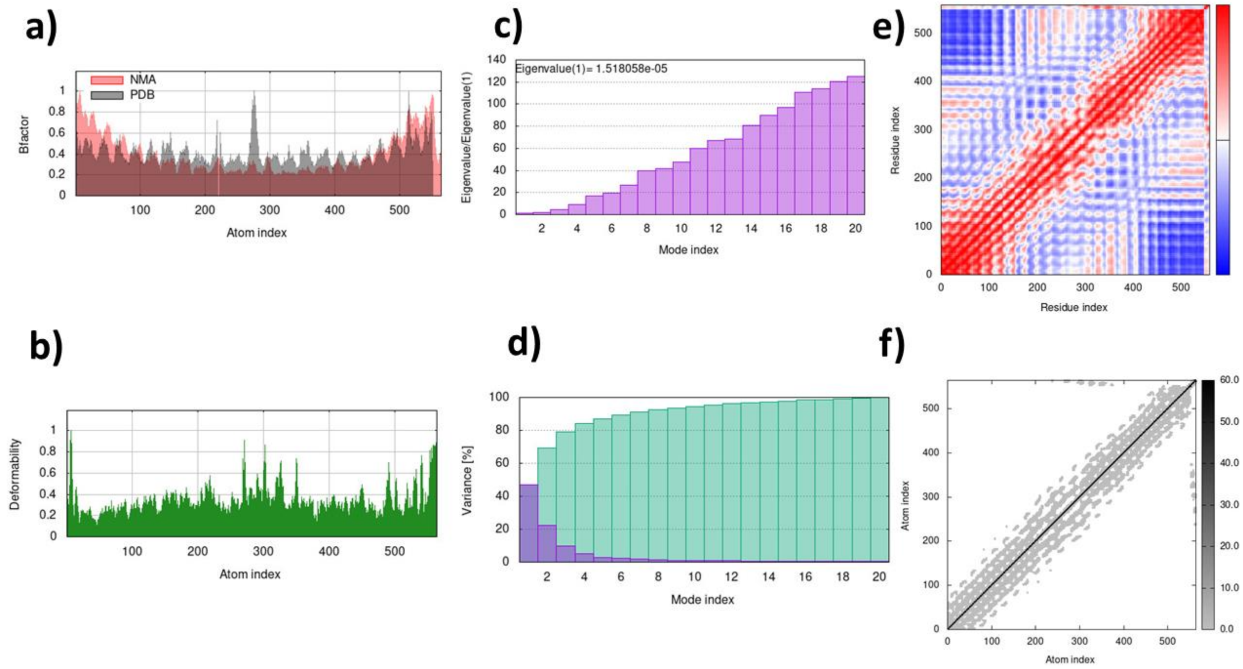

Molecular dynamics simulation of TLR2-PPpp113-266 complex as the antitubercular adjuvant. (a) B-factor, (b) Deformability, (c) Eigenvalues, (d) Variance, (e) Covariance matrix, (f) The elastic network map.

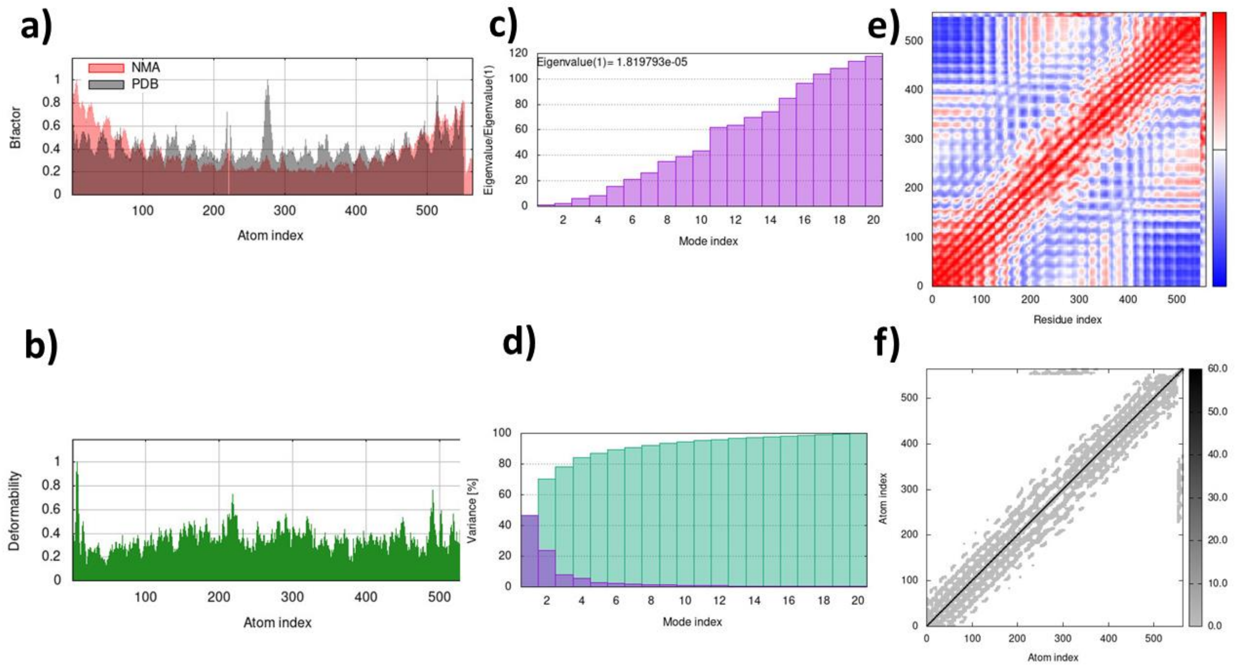

Molecular dynamics simulation of TLR2-LSsty1-174 complex as the antifungal adjuvant. (a) B-factor, (b) Deformability, (c) Eigenvalues, (d) Variance, (e) Covariance matrix, (f) The elastic network map.

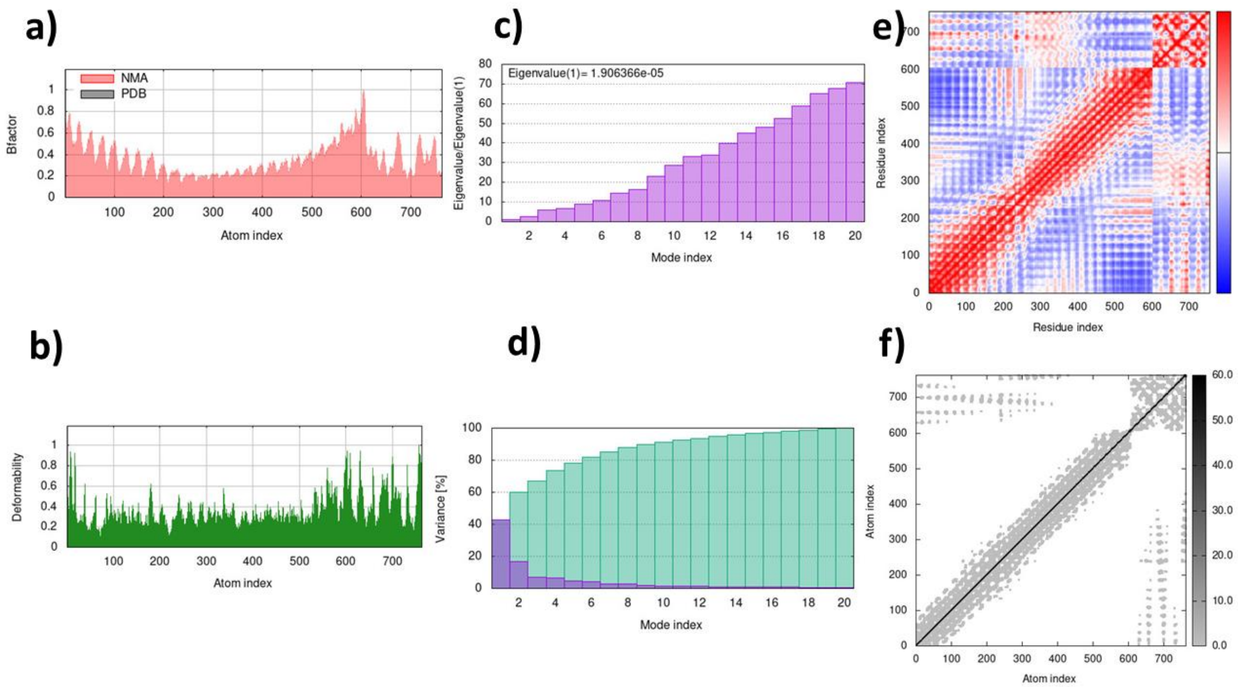

Molecular dynamics simulation of TLR4/MD2-SBsib-711 complex as the anticancer adjuvant. (a) B-factor, (b) Deformability, (c) Eigenvalues, (d) Variance, (e) Covariance matrix, (f) The elastic network map.
